# La_1–*x*_Sr_*x*_FeO_3−δ_ Perovskite Oxide Nanoparticles for Low-Temperature Aerobic Oxidation of Isobutane to *tert*-Butyl Alcohol

**DOI:** 10.1021/acsami.4c15585

**Published:** 2024-11-01

**Authors:** Masanao Yamamoto, Takeshi Aihara, Keiju Wachi, Michikazu Hara, Keigo Kamata

**Affiliations:** † Laboratory for Materials and Structures, Institute of Innovative Research, Tokyo Institute of Technology, Nagatsuta-cho 4259-R3-6, Midori-ku, Yokohama-city, Kanagawa 226-8501, Japan; ‡ Materials and Structures Laboratory, Institute of Integrated Research, Institute of Science Tokyo, Nagatsuta-cho 4259-R3-6, Midori-ku, Yokohama-city, Kanagawa 226-8501, Japan

**Keywords:** light alkane, selective oxidation, perovskite oxide, nanoparticle, iron

## Abstract

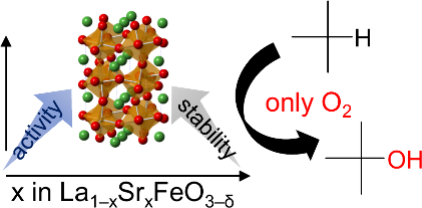

The development of reusable solid catalysts based on naturally abundant metal elements for the liquid-phase selective oxidation of light alkanes under mild conditions to obtain desired oxygenated products, such as alcohols and carbonyl compounds, remains a challenge. In this study, various perovskite oxide nanoparticles were synthesized by a sol–gel method using aspartic acid, and the effects of A- and B-site metal cations on the liquid-phase oxidation of isobutane to *tert*-butyl alcohol with molecular oxygen as the sole oxidant were investigated. Iron-based perovskite oxides containing Fe^4+^ such as BaFeO_3−δ_, SrFeO_3−δ_, and La_1–*x*_Sr_*x*_FeO_3−δ_ exhibited catalytic performance superior to those of other Fe^3+^- and Fe^2+^-based iron oxides and Mn-, Ni-, and Co-based perovskite oxides. The partial substitution of Sr for La in LaFeO_3_ significantly enhanced the catalytic performance and durability. In particular, the La_0.8_Sr_0.2_FeO_3−δ_ catalyst could be recovered by simple filtration and reused several times without an obvious loss of its high catalytic performance, whereas the recovered BaFeO_3−δ_ and SrFeO_3−δ_ catalysts were almost inactive. La_0.8_Sr_0.2_FeO_3−δ_ promoted the selective oxidation of isobutane even under mild conditions (60 °C), and the catalytic activity was comparable to that of homogeneous systems, including halogenated metalloporphyrin complexes. On the basis of mechanistic studies, including the effect of Sr substitution in La_1–*x*_Sr_*x*_FeO_3−δ_ on surface redox reactions, the present oxidation proceeds via a radical-mediated oxidation mechanism, and the surface-mixed Fe^3+^/Fe^4+^ valence states of La_1–*x*_Sr_*x*_FeO_3−δ_ nanoparticles likely play an important role in promoting C–H activation of isobutane as well as decomposition of *tert*-butyl hydroperoxide.

## 1 Introduction

**Figure 1:**
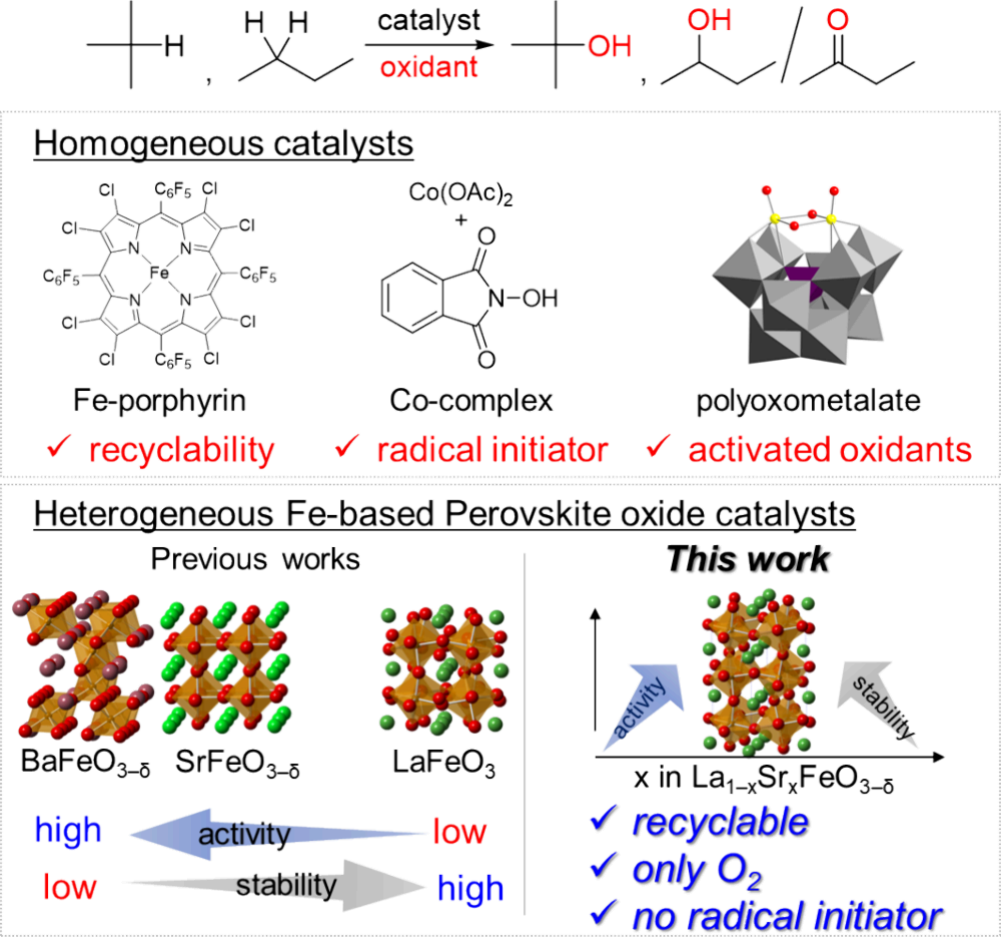
Catalytic oxidation of light alkanes to the corresponding oxygenated products.

Selective oxidation has received considerable attention not only in the production of useful oxygenated products (e.g., alcohols, aldehydes, ketones, carboxylic acids, epoxides, esters, and sulfoxides)^1−3^ but also in environmental chemistry (e.g., oxidative removal of volatile organic compounds).^4,5^ In particular, the development of a method for the oxyfunctionalization of light alkanes (C1–C4) as sources for the corresponding alkenes or their derivatives remains strongly desirable due to their availability and low cost and is a challenging subject of research.^6−9^ Catalytic gas-phase oxidative coupling, dehydrogenation, and O-/N-insertion reactions of light alkanes have been extensively studied, and some processes including the direct conversion of butanes into acetic acid, maleic anhydride, and butadiene and propane to acrylic acid have been commercialized.^6,7^ On the other hand, liquid-phase oxidation of light alkanes offers the advantage of suppressing the complete combustion of the alkanes to carbon oxides (CO_*x*_) due to milder reaction conditions than those in the gas phase.^9−19^ Although efficient catalytic systems such as halogenated metalloporphyrin,^16,19^ Os-/Fe-/Cu-/V-/Co-based complexes,^12−15,17,20^ metal–organic frameworks,^11^ and polyoxometalates^14^ have been reported for the oxidation of light alkanes, most of them are homogeneous and have drawbacks in the separation and recyclability of catalysts from the reaction mixtures (Figure 1). In addition, activated oxidants [hydrogen peroxide (H_2_O_2_),^14,18,20^ 2,6-dichloropyridine *N*-oxide,^15^ N_2_O,^11^ K_2_S_2_O_8_,^11^ etc.], radical initiators [*N*-hydroxyphthalimide,^12,17^*tert*-butyl hydroperoxide (TBHP),^11^ ditertiary butyl peroxide,^21^ etc.], and/or photoirradiation^18^ are typically required to obtain the desired products in high yields. Therefore, examples of recoverable and reusable heterogeneous catalysts with O_2_ as the sole oxidant are rare. In this work, the reaction scope begins with the oxidation of isobutane to *tert*-butyl alcohol (*t*-BuOH) which has multiple applications in the syntheses of pharmaceuticals, agrochemicals, and other fine chemicals^21^ and extends to the oxidation of *n*-butane into the corresponding alcohols and ketones.

C–H activation is a key step for the selective oxidation of alkanes.^1−3^ In biological and chemical oxidation, high-valent metal–oxo (M=O) complexes and photoexcited terminal oxo groups in polyoxometalates efficiently promote C–H activation, and it has been proposed that metal oxyl (M–O·) species play an important role in the potentially difficult oxidative conversion of substrates.^22,23^ On the other hand, some metal oxides with high (unusual) valence B-site metal cations provide ligand hole states, which affect electrical transport and magnetism.^24−26^ Notably, perovskite oxides ABO_3_ are promising materials due to their flexible structures/compositions and tunable physicochemical properties^27−29^ and have been extensively studied as catalysts for redox reactions such as gas-phase combustion of volatile organic compounds,^30^ oxidation of CO,^31^ and reduction of NO_*x*_,^32^ whereas liquid-phase selective C–H oxidation of alkanes has been scarcely reported despite similarities in active metal oxyl species between homogeneous systems and solid materials. During the course of the investigation on the unique catalysis by crystalline complex oxides,^33−39^ we reported for the first time that high-valent iron-based perovskite oxides such as BaFeO_3−δ_ and SrFeO_3−δ_ work as effective heterogeneous catalysts for the aerobic oxidation of adamantane and alkylarenes without the need for additives, whereas Fe^3+^- and Fe^2+^-containing oxides including LaFeO_3_ are completely inactive.^34^ On the other hand, LaFeO_3_ exhibited higher stability for the oxidative transformation of α-bromostyrene to phenacyl bromide than BaFeO_3−δ_ and SrFeO_3−δ_.^41^ However, in our preliminary examinations of the oxidation of isobutane, we found that high-valent iron-based perovskite oxides (BaFeO_3−δ_ and SrFeO_3−δ_) and LaFeO_3_ have drawbacks in reusability and C–H bond activation, respectively. These challenges motivated us to further develop the iron-based perovskite oxide catalysts through a multielement approach.

Herein, we focused on the partial substitution of La^3+^ in LaFeO_3_ with Sr^2+^ to achieve both stability and reactivity of iron-based perovskite oxides for liquid-phase oxidation reactions (Figure 1). Such a A-site substitution strategy has been developed to optimize a balance between activity and stability of Mn-, Fe-, and Co-based lanthanide perovskite oxides which exhibit high activity for gas-phase oxidation reactions;^40,41^ however, the application of liquid-phase oxidation of alkanes, especially using nanoparticle materials, has scarcely been reported. In this paper, we report that La_1–*x*_Sr_*x*_FeO_3−δ_ nanoparticles, which were synthesized by the sol–gel method using aspartic acid,^33−36,39^ can act as effective reusable heterogeneous catalysts for the aerobic oxidation of isobutane to *t*-BuOH using only O_2_. The partial substitution of Sr for La in LaFeO_3_ significantly enhanced the catalytic performance and durability, and La_0.8_Sr_0.2_FeO_3−δ_ exhibited high catalytic activity comparable to that of homogeneous catalysts. Although several heterogeneous catalysts, such as molybdenum oxide for the peroxidation of isobutane to TBHP^42^ and amorphous manganese oxide for the decomposition of TBHP to *t*-BuOH,^21^ have been reported, this study provides the first example of recoverable and reusable earth-abundant iron-based solid catalysts for the selective one-pot oxidation of isobutane to *t*-BuOH.

## 2 Experimental Section

### 2.1 Instruments

Characterization of the solid materials was performed using X-ray diffraction (XRD), infrared (IR) spectroscopy, thermogravimetry-differential thermal analysis (TG-DTA), inductively coupled plasma-atomic emission spectroscopy (ICP-AES), nitrogen adsorption–desorption, transmission electron microscopy (TEM), high-angle annular dark-field scanning transmission electron microscopy (HAADF-STEM), X-ray photoelectron spectroscopy (XPS), H_2_ temperature-programmed reduction (H_2_-TPR) analysis, and X-ray absorption spectroscopy as our previous reports.^33−39^ The details are described in the Supporting Information.

### 2.2 Synthesis of Perovskite Oxides

Perovskite oxide catalysts were synthesized by the sol–gel method using aspartic or malic acids in combination with metal acetates. A typical procedure for the iron-based La_0.8_Sr_0.2_FeO_3_ perovskite catalyst was synthesized by the amino acid-aided method as described in refs (40−46): La(OAc)_3_·1.5H_2_O (3.57 g, 10.4 mmol), Sr(OAc)_2_·0.5H_2_O (0.56 g, 2.6 mmol), Fe(OAc)_2_ (13 mmol), and l-aspartic acid (39 mmol) were dissolved in water (500 mL). The brown solution was evaporated to dryness at 60 °C. The resulting brown powder was dried at 240 °C for 2 h under vacuum to give a pale brown powder. The precursor was calcined at 650 °C for 5 h in air to obtain La_0.8_Sr_0.2_FeO_3−δ_ (2.61 g, 87% yield). The details for other perovskite oxides are shown in the Supporting Information.

### 2.3 Procedure for Catalytic Oxidation of Isobutane and *n*-Butane

Catalytic oxidation was conducted in a 13 mL autoclave reactor with a Teflon vessel containing a magnetic stirring bar. A typical procedure for catalytic oxidation of isobutane is as follows: La_0.8_Sr_0.2_FeO_3−δ_ (0.1 g), isobutane (0.2 MPa, 3.2 mmol), benzotrifluoride (PhCF_3_, 2 mL), and O_2_ (0.25 MPa) were taken. The amounts of isobutane introduced in the reactor were confirmed by the direct weight measurement using an advanced-level analytical/precision balance (A&D, GX-1603A). The reaction solution was heated at 110 °C for 24 h. After checking the residual pressure in the autoclave reactor, the products in the gas phase were transferred into a sampling bag and analyzed by GC-TCD with gaskuropack 54 and molecular sieve 5A columns. The products in the liquid phase were analyzed by GC-FID with a Stabilwax column. Yields and selectivities were calculated on the C4-basis. After the reaction, the catalyst was recovered by filtration, washed with PhCF_3_ (20 mL) and methanol (20 mL), and then dried at 110 °C for 12 h before recycling. The amounts of surface Fe species were estimated by assuming that the (001) plane is a surface structure because of the abundant population of Fe species (6.6 atoms per nm^2^) on the (001) plane. The amounts of surface Fe were estimated using this hypothesis, and the *S*_BET_ of La_0.8_Sr_0.2_FeO_3−δ_ was calculated to be 190 μmol g^–1^.

### 2.4 Procedure for Catalytic Decomposition of TBHP

Catalytic decomposition of TBHP was conducted in a 30 mL glass vessel containing a magnetic stirring bar. A typical procedure for catalytic decomposition was as follows: La_0.8_Sr_0.2_FeO_3−δ_ (50 mg), TBHP (0.5 mmol), PhCF_3_ (2 mL), Ar (0.1 MPa), and an internal standard (naphthalene) were charged into the reaction vessel. The reaction solution was heated to 50 °C and periodically analyzed using GC.

## 3 Results and Discussion

### 3.1 Characterization of Iron-Based Perovskite Oxide Nanoparticles

**Figure 2:**
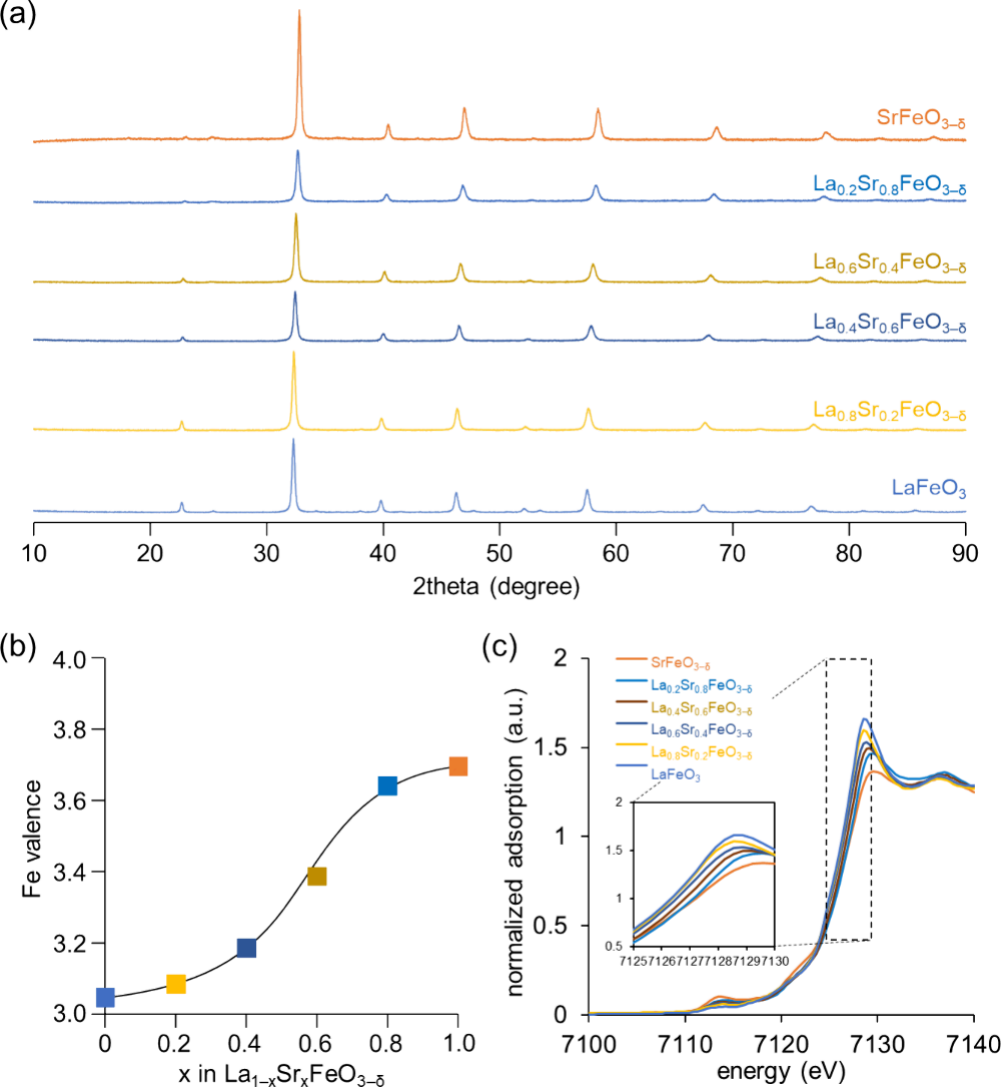
(a) XRD patterns of La_1–*x*_Sr_*x*_FeO_3−δ_. (b) Fe valence determined by iodometry. (c) XANES spectra of La_1–*x*_Sr_*x*_FeO_3−δ_.

**Figure 3:**
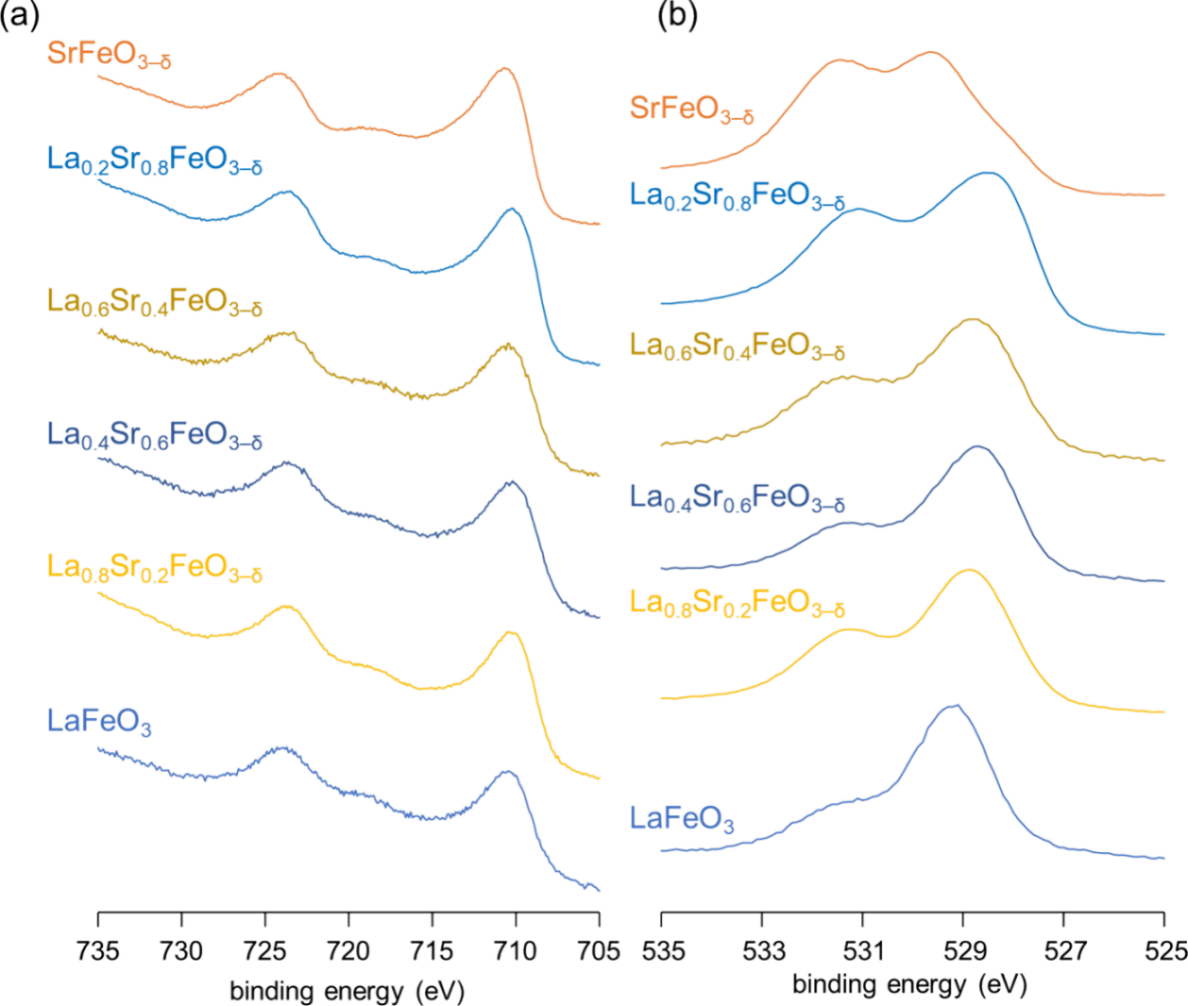
(a) Fe 2p and (b) O 1s XPS spectra of La_1–*x*_Sr_*x*_FeO_3−δ_.

Various metal-oxide nanoparticles including iron-based perovskite oxides were synthesized by our sol–gel method using dicarboxylic acids such as aspartic acid or malic acid.^33−36,39^ In the synthesis of La_0.8_Sr_0.2_FeO_3−δ_, the precursor was completely amorphous, as indicated by the absence of XRD peaks (Figure S1). The IR spectrum of the precursor shows the peaks at 1386 and 1541 cm^–1^, which are assignable to the symmetric and asymmetric stretching vibrations, respectively (Figure S2).^43^ Since the large separation (155 cm^–1^) of these two peaks indicates the bridging bidentate bonding, aspartate anions linked the metal cations to form the amorphous precursor. Furthermore, TG-DTA curves of the precursor had exothermic peaks with weight losses around 280 and 420 °C, suggesting the decomposition of the precursor below 650 °C (Figure S3). Figure 2a shows the XRD patterns for La_1–*x*_Sr_*x*_FeO_3−δ_ (*x* = 0, 0.2, 0.4, 0.6, 0.8, and 1.0). La_1–*x*_Sr_*x*_FeO_3−δ_ is composed of a general perovskite oxide framework with corner-sharing FeO_6_ octahedral units in contrast to hexagonal BaFeO_3−δ_ which consists of face-sharing dimeric Fe_2_O_9_ units linked by single corner-sharing FeO_6_ units along the *c* axis (Figure 1). The XRD peaks for orthorhombic LaFeO_3_ (space group *Pbnm*) shift toward higher angle with increasing Sr content (*x*) due to the lattice size reduction, and the perovskite structure changes to cubic. Since the ionic radius of La^3+^ (1.34 Å) is smaller than that of Sr^2+^ (1.44 Å),^44−46^ such a lattice size change is likely caused by the oxidation of Fe^3+^ (0.645 Å) to Fe^4+^ (0.585 Å). The molar ratios of La, Sr, and Fe were in good agreement with the stoichiometry based on the elemental analysis of La_1–*x*_Sr_*x*_FeO_3−δ_ using ICP-AES (Table S1). The effect of Sr substitution on the oxidation state of iron in La_1–*x*_Sr_*x*_FeO_3−δ_ was examined by iodometry, X-ray absorption near edge structure (XANES) and XPS. The bulk oxidation states of iron obtained from the iodometry increase with increasing Sr substitution (3.05, 3.09, 3.19, 3.39, 3.64, and 3.70 for *x* = 0, 0.2, 0.4, 0.6, 0.8, and 1.0, respectively), while the valence is smaller than that expected for stoichiometric AFeO_3_ probably due to the presence of oxygen vacancies (Figure 2b). Figure 2c shows the Fe *K*-edge positions for the normalized XANES spectra of La_1–*x*_Sr_*x*_FeO_3−δ_. A positive shift of the absorption edge position around 7127 eV is observed as the Sr content increases, which indicates that the iron valence also increases.^47,48^ In addition, the pre-edge peak intensity at around 7115 eV increases with an increase in the Sr content. The pre-edge feature is attributed to a forbidden transition from the 1s to 3d state, which is allowed by mixing between the Fe 3d and O 2p states. Therefore, the increase in the intensity of these pre-edge peaks is related to an increase in the symmetry of the FeO_6_ octahedron. This result is in accordance with the XRD results in that an orthorhombic LaFeO_3_ structure changes into a less distorted cubic SrFeO_3−δ_ structure by Sr substitution. The surface structure of La_1–*x*_Sr_*x*_FeO_3−δ_ was evaluated by XPS analysis, and Figure 3 shows the Fe 2p and O 1s XPS spectra for La_1–*x*_Sr_*x*_FeO_3−δ_ including the deconvolution results (Figure S4). The Fe 2p XPS peaks for La_1–*x*_Sr_*x*_FeO_3−δ_ shift to higher binding energy with increasing Sr content (Figure 3a). The surface oxidation states of iron estimated based on the XPS results (3.14, 3.27, 3.30, 3.42, 3.54, and 3.72 for *x* = 0, 0.2, 0.4, 0.6, 0.8, and 1.0, respectively) also increase with increasing Sr substitution in a manner similar to that of the bulk oxidation states. In addition, the O 1s XPS spectra also show an increase in the ratio of peak intensity for adsorbed oxygen species to lattice oxygen species (Figure 3b). All these results suggest that the valence of Fe and the amount of oxygen vacancies increase as the Sr content increases, in agreement with previous reports.^44−47^

**Figure 4:**
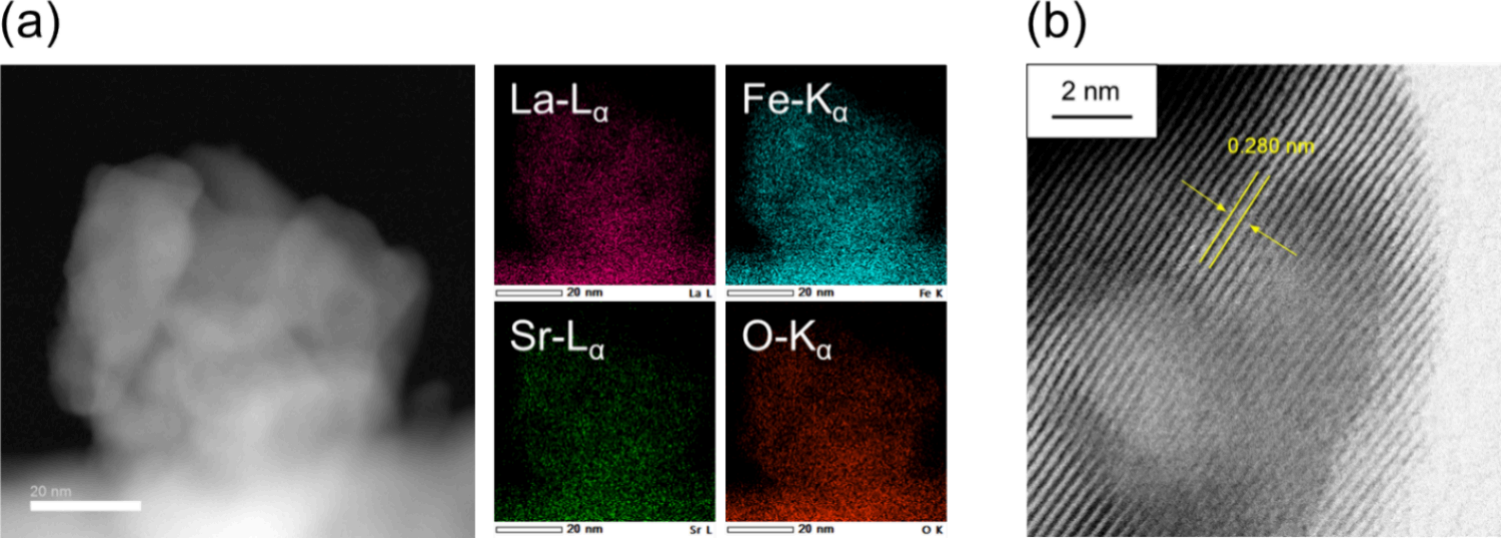
(a) STEM image and the corresponding EDS elemental mapping and (b) TEM image of La_0.8_Sr_0.2_FeO_3−δ_.

In the case of our sol–gel methods using aspartic or malic acid, a low-density amorphous precursor decomposed at relatively low temperature to form crystalline nanoparticles with a high surface area in comparison with other sol–gel methods such as the polymerized-complex and Pechini methods via carbonaceous precursors.^33−36,39^ The specific surface areas (*S*_BET_) for the present La_1–*x*_Sr_*x*_FeO_3−δ_ catalysts obtained by the calcination of amorphous precursors even at relatively low temperature (650 °C) were in the range of 17 to 20 m^2^ g^–1^, which were higher than those for previously reported synthetic methods that required calcination at higher temperatures, e.g., the Pechini method (2.0–11.5 m^2^ g^–1^, 800–900 °C),^44,46^ the citrate method (9.0–13.8 m^2^ g^–1^, 800 °C),^49^ the solution combustion method (2.3–6.7 m^2^ g^–1^, 900 °C),^50^ and the coprecipitation method (0.5–11.4 m^2^ g^–1^, 780–1050 °C).^51^ A STEM image and element maps obtained by energy-dispersive X-ray spectroscopy (EDS) for La_0.8_Sr_0.2_FeO_3−δ_ are shown in Figure 4a. The formation of aggregates of spherical nanoparticles with estimated particle sizes of ca. 20–40 nm and their distribution agreed well with the grain sizes of La_1–*x*_Sr_*x*_FeO_3−δ_ (20–30 nm) calculated from the diffraction peaks around 32° using the Scherrer equation. The EDS mapping indicates a uniform distribution of constituent elements (La, Sr, Fe, and O) in the nanoparticles, in good agreement with the XRD results. Figure 4b shows the TEM image of La_0.8_Sr_0.2_FeO_3−δ_. Clear lattice fringes with *d*-spacings of 0.28 nm assignable to the (020) planes of the orthorhombic structure were observed in the particles.

### 3.2 Liquid-Phase Catalytic Oxidation of Isobutane and *n*-Butane with Molecular Oxygen

**Figure 5:**
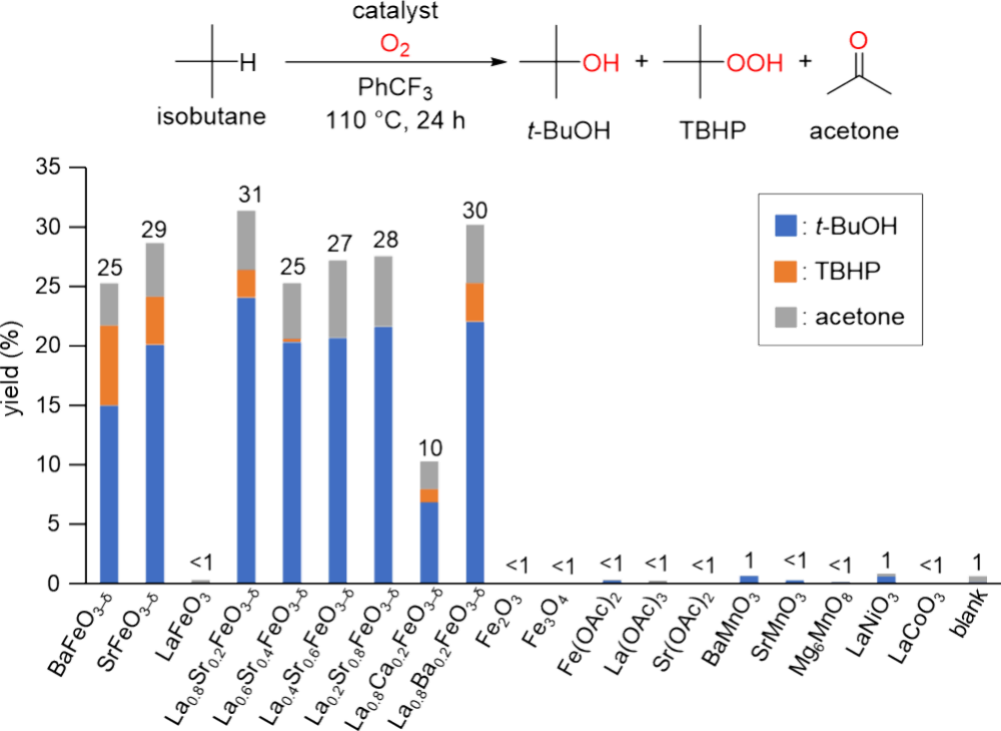
Effect of catalysts on the oxidation of isobutane with O_2_. Reaction conditions: Catalyst (0.1 g), isobutane (0.2 MPa), O_2_ (0.25 MPa), and PhCF_3_ (2 mL), 110 °C, 24 h. The details are shown in Table S2.

We focused on the selective oxidation of isobutane to *t*-BuOH as a direct alternative to conventional processes such as coproduction during the propylene oxide process and homogeneously- and heterogeneously-catalyzed hydration of isobutylene.^52^*t*-BuOH is an important alcohol that acts an alkylating agent for phenols^53^ and biodiesel additives^54^ and as a starting material for isobutylene.^55^ First, liquid-phase oxidation of isobutane (0.2 MPa) with O_2_ (0.25 MPa) as the sole oxidant at 110 °C was carried out in the presence of various metal-oxide catalysts (Figure 5, Table S2). The three main products were *t*-BuOH, TBHP, and acetone. In the absence of a catalyst, the reaction did not proceed at all. We previously reported that high-valent iron-containing perovskite oxides such as BaFeO_3−δ_ and SrFeO_3−δ_ were effective for the oxidation of adamantane with atmospheric-pressure O_2_ in sharp contrast to Fe^3+^- and Fe^2+^-containing iron oxides.^35^ BaFeO_3−δ_ and SrFeO_3−δ_ similarly catalyzed the aerobic oxidation of isobutane to give the products in 25 and 29% total yields, respectively, whereas LaFeO_3_ was inactive for the oxidation. On the other hand, La_1–*x*_Sr_*x*_FeO_3−δ_ also exhibited high catalytic oxidation activity similar to BaFeO_3−δ_ and SrFeO_3−δ_. In the case of La_0.8_Sr_0.2_FeO_3−δ_, the total yield reached up to 31% with the selectivity for *t*-BuOH (71%), TBHP (7%), and acetone (19%), and the formation of CO and CO_2_ in the gas-phase was small (entry 1, Table 1). In addition, isobutane oxidation with air as the oxidant was conducted using the La_0.8_Sr_0.2_FeO_3−δ_ catalyst since industrial processes operate above the upper flammability limit, and air is favorable as a more cost-effective oxidant compared to pure O_2_ (entry 4, Table 1). Although the yield slightly decreased, it was confirmed that the reaction proceeded without any change in the selectivity. From the effect of the A-site metal cations in La_0.8_A_0.2_FeO_3−δ_, Sr substitution was superior to the use of Ca or Ba in terms of yield and/or selectivity. C–H oxidation of isobutane did not occur with other iron oxides (LaFeO_3_, Fe_2_O_3_, and Fe_3_O_4_), catalyst precursors [Fe(OAc)_2_, La(OAc)_3_, and Sr(OAc)_2_], perovskite oxides (SrMnO_3_, BaMnO_3_, LaNiO_3_, and LaCoO_3_), or the murdochite-type oxide Mg_6_MnO_8_, the latter of which efficiently catalyzes the aerobic oxidation of alkylarenes under mild conditions.^39^

**Table 1:**
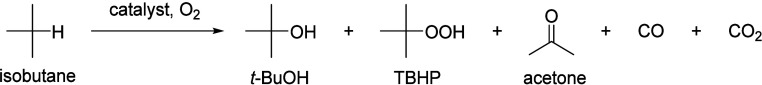
Effect of Oxidants on the Oxidation of Isobutane over La_0.8_Sr_0.2_FeO_3−δ_^a^

entry	oxidant	temperature (°C)	total yield (%)	selectivity (%)				
				*t*-BuOH	TBHP	acetone	CO	CO_2_
1	O_2_ (0.25 MPa)	110	31	71	7	19	<1	2
2	O_2_ (0.25 MPa)	60	12	84	9	6	<1	1
3	O_2_ (0.50 MPa)	110	55	76	7	15	<1	2
4	air (1.0 MPa)	110	16	69	1	30	b	b

a Reaction conditions: La_0.8_Sr_0.2_FeO_3−δ_ (0.1 g), isobutane (0.2 MPa, 3.2 mmol), PhCF_3_ (2 mL), 24 h.

b The products in the gas phase were not measured.

**Figure 6:**
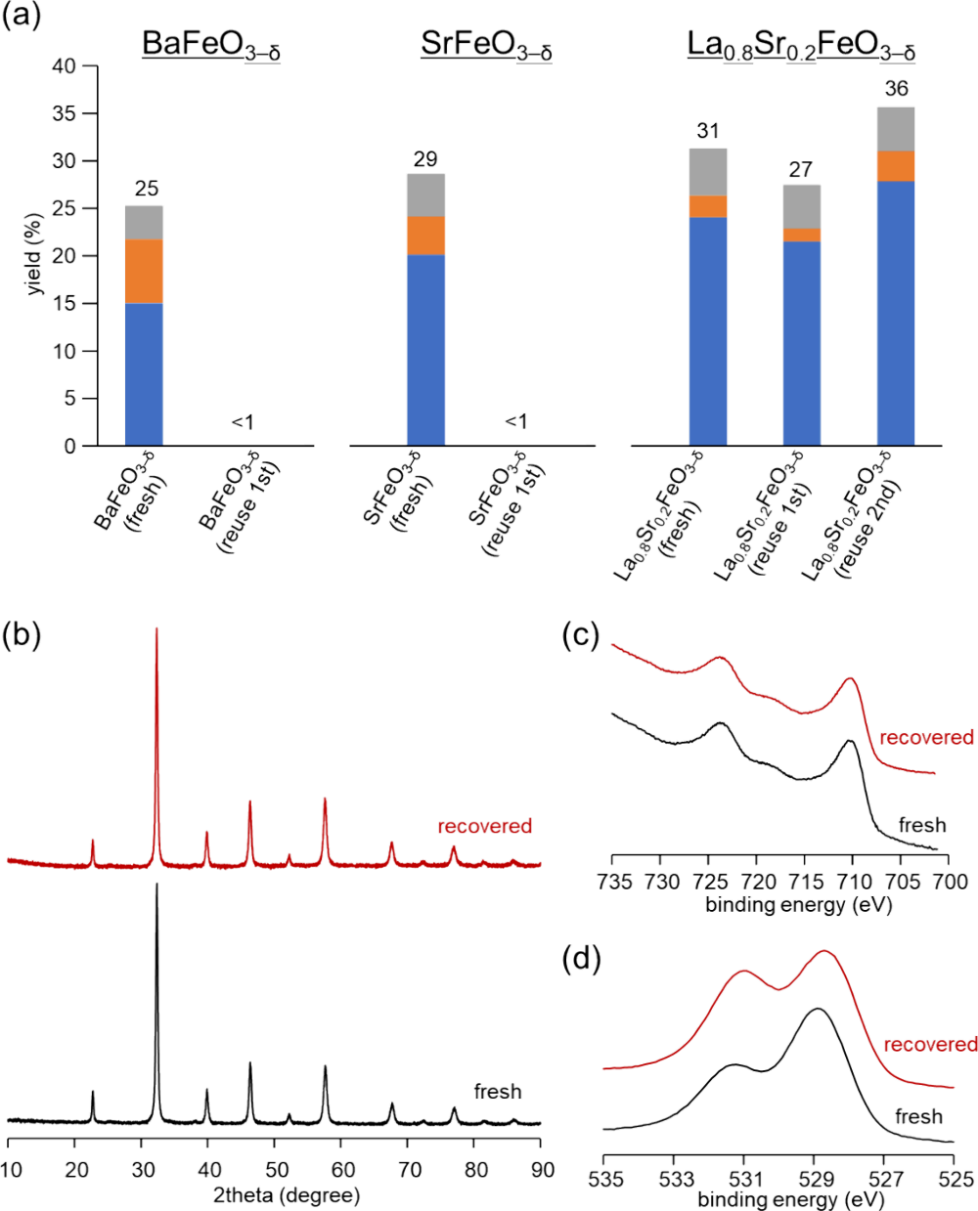
(a) Recycling of La_0.8_Sr_0.2_FeO_3−δ_, SrFeO_3−δ_, and BaFeO_3−δ_ for the oxidation of isobutane with O_2_. Reaction conditions are the same as those in Figure 5. The details are shown in Table S3. (b) XRD patterns, (c) XPS Fe 2p and (d) XPS O 1s spectra of fresh and recovered La_0.8_Sr_0.2_FeO_3−δ_.

Next, the reusability of iron-based perovskite oxides, including La_0.8_Sr_0.2_FeO_3−δ_, BaFeO_3−δ_, and SrFeO_3−δ_ for the present oxidation of isobutane, was investigated. After the oxidation reaction under the conditions in Figure 6a and Table S3, the used catalysts could readily be recovered from the reaction mixture by simple filtration. The recovered BaFeO_3−δ_ and SrFeO_3−δ_ catalysts were almost inactive. On the other hand, the recovered La_0.8_Sr_0.2_FeO_3−δ_ catalyst could be reused without significant change in the total yield or selectivity, which indicated the durability of La_0.8_Sr_0.2_FeO_3−δ_. The elution of active metal species into the reaction solution was not indicated by ICP-AES (La, Sr, Fe: <0.01% with respect to fresh La_0.8_Sr_0.2_FeO_3−δ_). There was no significant difference in the XRD patterns and XPS spectra for the fresh and recovered La_0.8_Sr_0.2_FeO_3−δ_ catalyst, which indicated the durability of La_0.8_Sr_0.2_FeO_3−δ_ (Figure 6b,c). In contrast, significant bulk and/or surface structure changes were confirmed by XRD patterns and XPS spectra for the recovered BaFeO_3−δ_, which suggested that the catalyst instability prevented their reuse (Figures S5 and S6). The XRD pattern for the recovered BaFeO_3−δ_ indicated the formation of an oxygen-deficient phase which is similar to BaFeO_2.667_.^56^ While no significant difference in the XRD patterns between fresh and used SrFeO_3−δ_ catalysts was observed (Figure S5), large differences in the Fe 2p and O 1s XPS spectra were observed; thus, the surface structure of SrFeO_3−δ_ changed during the reaction. In order to investigate the surface structure of SrFeO_3−δ_ in more detail, the deconvolution of XPS spectra in the Fe 2p and O 1s regions was conducted (Figure S6 and Table S4). In the Fe 2p XPS spectra of SrFeO_3−δ_, the area ratio of the peak attributed to Fe^4+^ to that to Fe^3+^ (i.e., surface Fe^4+^/Fe^3+^ ratio) decreased after the reaction (from 72/28 to 58/42).^57^ In addition, the peak intensity of adsorbed oxygen and/or water in the O 1s spectrum of recovered SrFeO_3−δ_ significantly increased with the appearance of a new peak around 533 eV which is assignable to CO_3_^2–^ species. While a decrease in the surface Fe^4+^/Fe^3+^ ratio (from 46/54 to 35/65) was observed in BaFeO_3−δ_, the changes in the surface Fe^4+^/Fe^3+^ ratio (from 27/73 to 32/68) and the content of adsorbed oxygen species (from 41 to 47%) were negligible in the case of La_0.8_Sr_0.2_FeO_3−δ_. Based on these observations, the irreversible change from Fe^4+^ to Fe^3+^ species would occur after the reaction, resulting in the poor reusability of SrFeO_3−δ_ and BaFeO_3−δ_. Therefore, the surface Fe^4+^ species plays a crucial role in the reusability of La_0.8_Sr_0.2_FeO_3−δ_. Since the above-mentioned oxidation reactions were carried out in a fluorinated solvent of PhCF_3_, we investigated the possible use of environmentally compatible solvents (Figure S7a).^58^ Chlorinated benzenes also gave the products in high yields, but nonpolar toluene and *n*-octane were not as effective because they themselves were oxidized during the reaction. Although the reaction did not proceed in aprotic polar solvents, the oxidation proceeded more efficiently in tertiary alcohol and ester solvents. The total product amount using ethyl acetate (EtOAc) was comparable to that using PhCF_3_, and the La_0.8_Sr_0.2_FeO_3−δ_ catalyst recovered after the reaction in EtOAc could be reused without a loss of catalytic performance or structure (Figure S7b,c). Moreover, in the absence of the La_0.8_Sr_0.2_FeO_3−δ_ catalyst or isobutane, no oxidation products were observed (Figure S8). These results confirmed that La_0.8_Sr_0.2_FeO_3−δ_ promoted the present oxidation reaction in ethyl acetate and that ethyl acetate did not function as an initiator or a sacrificial solvent.

**Figure 7:**
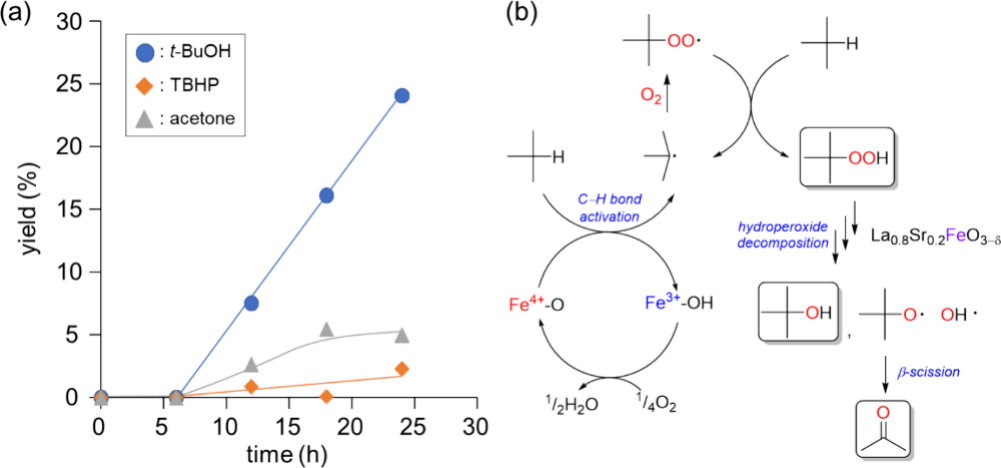
(a) Time course for the oxidation of isobutane with O_2_ over La_0.8_Sr_0.2_FeO_3−δ_. Reaction conditions are the same as those in Figure 5. (b) Proposed reaction mechanism over La_0.8_Sr_0.2_FeO_3−δ_.

Figure 7a shows the time course of the oxidation of isobutane using the La_0.8_Sr_0.2_FeO_3−δ_ catalyst. The oxidation reaction smoothly proceeded with an induction period of about 6 h, and the yield of *t*-BuOH increased linearly with the coproduction of acetone. The formation of TBHP was not as significant, which indicated the fast decomposition of TBHP to products, such as *t*-BuOH (as described below). The formation of acetone would be caused by C–C bond cleavage (β-scission) from a possible alkoxy radical intermediate. In addition, the oxidation of isobutane did not proceed under an Ar atmosphere, which confirmed that La_0.8_Sr_0.2_FeO_3−δ_ did not function as a stoichiometric oxidant but as a catalyst. Similar reactivity was observed for BaFeO_3−δ_, where a radical-mediated oxidation mechanism has been proposed.^34,35^ The addition of radical scavengers [2,6-di-*tert*-butyl-4-methylphenol (BHT), 2,2,6,6-tetramethylpiperidine 1-oxyl free radical (TEMPO), and *p*-benzoquinone (0.3 equiv with respect to isobutane)] completely suppressed the reaction progress (Table 2), indicating that *tert*-butyl radical (R·) and *tert*-butyl peroxy radical (ROO·) are likely involved in the present system.^59−61^ When 2-propanol was used as a scavenger of hydroxyl radical (OH·) to the reaction mixture, the formation of *t*-BuOH was completely suppressed, whereas acetone was formed likely due to the oxidation of 2-propanol. Therefore, OH· formed by the decomposition of TBHP would also be involved; thus, the present oxidation likely proceeds via the radical-mediated mechanism (Figure 7b) in a similar manner to that for previously reported systems.^16,19^

**Table 2:**
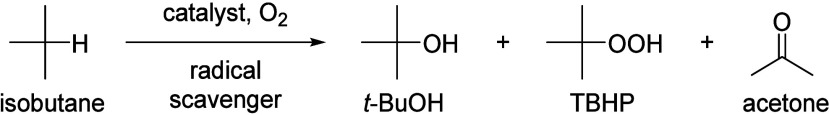
Effect of Radical Scavengers on the Oxidation of Isobutane over La_0.8_Sr_0.2_FeO_3−δ_^a^

entry	radical scavenger	total yield (%)	selectivity (%)		
			*t*-BuOH	TBHP	acetone
1	none	31	71	7	19
2	BHT	<1			
3	TEMPO	<1			
4	*p*-benzoquinone	<1			
5	2-propanol	3	<1	<1	>99

a Reaction conditions: La_0.8_Sr_0.2_FeO_3−δ_ (0.1 g), isobutane (0.2 MPa, 3.2 mmol), radical scavenger (1 mmol), O_2_ (0.25 MPa), and PhCF_3_ (2 mL), 110 °C, 24 h.

**Figure 8:**
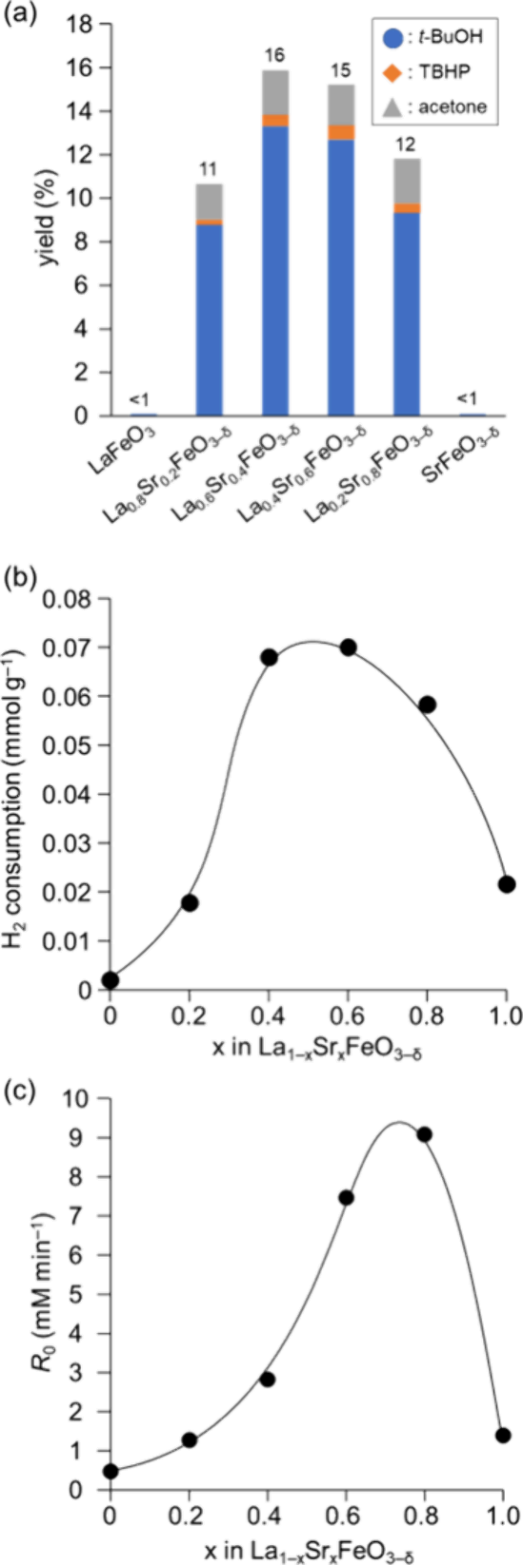
(a) Effect of Sr substitution for the oxidation of isobutane with O_2_ over La_1–*x*_Sr_*x*_FeO_3−δ_. Reaction conditions: Catalyst (0.1 g), isobutane (0.2 MPa), O_2_ (0.25 MPa), and PhCF_3_ (2 mL), 80 °C, 24 h. The details are shown in Table S5. Plots of (b) H_2_ consumption and (c) reaction rates for the decomposition of TBHP against Sr content.

To investigate the effect of Sr substitution in La_1–*x*_Sr_*x*_FeO_3−δ_ in more detail, we carried out the oxidation of isobutane under mild conditions. Figure 8a shows the relationship between the catalytic activity for oxidation at 80 °C and the Sr content (Table S5). While there is no substantial difference in the catalytic activity at 110 °C (Figure 5), a volcano-type relationship between the oxidation activity at 80 °C and the Sr content is observed. A similar volcano trend with increasing Sr contents has been observed in some cases such as high-temperature gas-phase reactions of CO and methane and electrochemical oxygen evolution/reduction reactions,^46,62^ and it has been proposed that the valence of B-site metal cations, the oxygen vacancy/mobility/activation, and the metal–oxygen covalency contribute to the high reactivity of perovskite oxides.^31,63−65^ In our cases, this order of Sr contents cannot be simply explained by an increase in the valence of surface and bulk iron species, the amounts of adsorbed oxygen species, and the surface (La + Sr)/Fe molar ratio (Figure S9). Thus, H_2_-TPR measurements were performed to confirm the oxidizing ability of surface oxygen species of La_1–*x*_Sr_*x*_FeO_3−δ_ (Figure S10). In the previously reported H_2_-TPR profiles of La_1–*x*_Sr_*x*_FeO_3−δ_, partial substitution of Sr for La in LaFeO_3_ increases the peak intensity attributed to the reduction of Fe^4+^ to Fe^3+^ at around 400–500 °C but shifts the reduction peak to higher temperature^54,55^; however, the catalytic activity has been mainly discussed based on the intensity and temperature of reduction peaks. We have reported a good correlation between the H_2_-consumption estimated from the initial reduction of H_2_-TPR profiles and the catalytic activity for several liquid-phase oxidations because liquid-phase reactions are typically carried out at relatively low temperatures.^33−39^ Therefore, H_2_ consumption below 200 °C and/or the onset reduction temperature were estimated from the H_2_-TPR profiles, and these parameters show similar volcano-type relationships with respect to the Sr content (Figure 8b). Such a dependence for La_1–*x*_Sr_*x*_FeO_3−δ_ has also been reported in NH_3_ oxidation and chemical-looping steam methane reforming systems, and it has been reported that the oxidizing power of Fe^4+^ species as well as the oxygen mobility are important factors for determining the gas-phase oxidation performance.^66,67^ In addition, the enthalpies of formation of La_1–*x*_Sr_*x*_FeO_3−δ_ from oxides increase as the Sr contents increase,^68^ and a balance between the oxidizing power and stability has been proposed.^62^ In the present oxidation of isobutane, not only C–H bond activation of isobutane with Fe^4+^ species but also regeneration of generated oxygen vacancies by O_2_ would be essential to catalytically promote the reaction. The low stability and reusability of BaFeO_3−δ_ and SrFeO_3−δ_ including the XRD and XPS results would also support this idea in the present oxidation. Furthermore, the difference in the amount of Sr substitution greatly affected another important reaction step, the decomposition of TBHP, and the optimal amount of Sr was confirmed (Figure 8c). The decomposition of hydroperoxides is accelerated by the redox reaction of metal species^69^; thus, the present surface-mixed Fe^3+^/Fe^4+^ valence states probably promote C–H activation of isobutane as well as the decomposition of TBHP (Figure 7b), which results in the high catalytic performance of La_1–*x*_Sr_*x*_FeO_3−δ_ nanoparticles for aerobic oxidation of isobutane.

**Figure 9:**
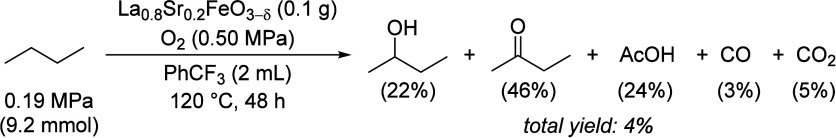
Liquid-phase oxidation of *n*-butane over La_0.8_Sr_0.2_FeO_3−δ_. The values in parentheses are the selectivity to each product.

La_0.8_Sr_0.2_FeO_3−δ_ efficiently catalyzed the oxidation of isobutane even at 60 °C (entry 2, Table 1), and 12% total yield was obtained with selectivity for *t*-BuOH (84%), TBHP (9%), and acetone (6%). In contrast, high reaction temperatures (∼130 °C), excess amounts of specific additives/oxidants, and high O_2_ pressures (∼3.5 MPa) were typically required for previously reported homogeneously catalyzed systems (Table S6).^12−20,22,23^ In addition, the total yield reached 55% for the oxidation of isobutane under oxygen-rich conditions (entry 3, Table 1), and the turnover number based on surface Fe species reached 93. In this case, 43% yield *t*-BuOH was higher than or comparable to those for homogeneous aerobic oxidation systems such as halogenated metalloporphyrin complexes (14–22%)^16,19^ (Table S6). On the other hand, the *t*-BuOH formation rate per catalyst weight of La_0.8_Sr_0.2_FeO_3−δ_ (0.57 mmol g-cat^–1^ h^–1^) was comparable to that of the polyoxometate/H_2_O_2_ system (0.62) but much lower than those of other homogeneous catalytic systems based on O_2_ and other activated oxidants in combination with radical initiators (6.2–2700) because of their all-catalytic sites accessible to substrates and oxidants. In particular, the electron-deficient iron porphyrin catalysts are effective for the liquid-phase oxidation of light alkanes, and decomposition of porphyrin-based catalyst has been reported.^19^ The present La_0.8_Sr_0.2_FeO_3−δ_ system is also applicable to the oxidation of *n*-butane with inert secondary C–H bonds to give 2-butanol, ethyl methyl ketone, and acetic acid selectively with a total yield of 4% (Figure 9). This study provides the first reported example of a reusable solid catalyst as an earth-abundant iron oxide for the aerobic oxidation of isobutane and *n*-butane without radical initiators.

## 4 Conclusions

In conclusion, iron-based perovskite oxide nanoparticles La_1–*x*_Sr_*x*_FeO_3−δ_ synthesized by the amino acid-aided method could heterogeneously catalyze the aerobic oxidation of isobutane to *t*-BuOH without the need for radical initiators under mild conditions. The activity of La_0.8_Sr_0.2_FeO_3−δ_ was much higher than those of Fe^3+^- and Fe^2+^-based iron oxides and Mn-, Ni-, and Co-based perovskite oxides, and the recovered catalyst could be reused without a significant loss of catalytic performance. In comparison with iron-based perovskite oxides of BaFeO_3−δ_ and SrFeO_3−δ_ containing Fe^4+^, the substitution effect of the A-site metal cations made it possible to introduce active and stable high-valent iron species, enabling a new approach to liquid-phase lower alkane oxidation using perovskite oxides. On the basis of mechanistic studies, the present oxidation likely proceeds via a radical-mediated oxidation mechanism, and surface redox properties likely contribute to the enhancement of C–H activation of isobutane as well as decomposition of TBHP. We have successfully developed a new solid catalyst that exhibits higher catalytic activity than previously reported homogeneous catalysts including halogenated metalloporphyrin catalysts. This study provides promising synthesis routes for *t*-BuOH and acetone using direct oxidation of isobutane without involving conventional coproduction processes. Furthermore, the applicability of the present catalytic system to the oxidation of *n*-butane offered valuable guidelines for catalyst design in the oxidation of linear alkanes.

## Supporting Information Available

Experimental details, comparison of previously reported oxidation systems, ICP-AES results, XPS area ratio, detailed oxidation data, XRD patterns, XPS spectra, solvent effect, TG-DTA curves, blank experiment, FT-IR spectra, and H_2_-TPR profiles (PDF)
