# Supplementary material for: La1–xSrxFeO3−δ Perovskite Oxide Nanoparticles for Low-Temperature Aerobic Oxidation of Isobutane to tert-Butyl Alcohol
Source: ACS Appl Mater Interfaces. 2024 Nov 1;16(45):62244–53. doi: 10.1021/acsami.4c15585 (PMC11565478; doi:10.1021/acsami.4c15585)
Supplement: Supplementary file 1 — am4c15585_si_001.pdf [file am4c15585_si_001.pdf]

Supporting Information for

$La_{1-x}Sr_xFeO_{3-\delta}$ Perovskite Oxide Nanoparticles for Low-Temperature Aerobic Oxidation of Isobutane to *tert*-Butyl Alcohol

Masanao Yamamoto, Takeshi Aihara, Keiju Wachi, Michikazu Hara, Keigo Kamata*

Laboratory for Materials and Structures, Institute of Innovative Research, Tokyo Institute of Technology, Nagatsuta-cho 4259-R3-6, Midori-ku, Yokohama-city, Kanagawa, 226-8501, Japan. E-mail: kamata.k.ac@m.titech.ac.jp
Materials and Structures Laboratory, Institute of Integrated Research, Institute of Science Tokyo, Nagatsuta-cho 4259-R3-6, Midori-ku, Yokohama-city, Kanagawa, 226-8501, Japan. E-mail: kamata@msl.iir.isct.ac.jp

Experimental section

Materials

Reagents such as $La(OAc)_3 \cdot 1.5H_2O$ (Kanto Chemical), $Sr(OAc)_2 \cdot 0.5H_2O$ (Kanto Chemical), $Fe(OAc)_2$ (Wako Chemical, 28 wt% Fe, determined by ICP-AES), aspartic acid (Kanto Chemical), Fe_3O_4 (Aldrich), Fe_2O_3 (Aldrich), *tert*-butyl alcohol (Aldrich), *tert*-butyl hydroperoxide (Aldrich, 70% decane solution), acetone (Kanto Chemical), 2-butanol (TCI), 2-butanone (Kanto Chemical), and acetic acid (Kanto Chemical) were purchased and used as-received. Distilled water was produced in-house using a water purifier (Merck Millipore Elix Essential UV 3). Solvents (benzotrifluoride (TCI), *tert*-amyl alcohol (TCI), *N*,*N*-dimethylformamide (TCI), acetonitrile (TCI), benzonitrile (TCI), *N*,*N*-dimethylacetamide (TCI), dichlorobenzene (TCI), anisole (TCI), butyl acetate (TCI), sulfolane (TCI), chlorobenzene (Kanto Chemical), toluene (Kanto Chemical), *n*-octane (Kanto Chemical), ethyl acetate (Kanto Chemical), were pretreated with molecular sieves (3A) that were evacuated at 280 °C for 3 h.[S1] Isobutane (99.9%), *n*-butane (99.8%), and oxygen (99.5%) were used as-received for the catalytic oxidation of butane.

Instruments

The characterization of the solid materials were investigated using X-ray diffraction (XRD), inductively coupled plasma-atomic emission spectroscopy (ICP-AES), nitrogen adsorption–desorption, transmission electron microscopy (TEM), high-angle annular dark-field scanning transmission electron microscopy (HAADF-STEM), iodometric titration, X-ray photoelectron spectroscopy (XPS) H_2 temperature-programmed reduction (H_2-TPR) analysis, and X-Ray absorption spectroscopy (XAS) using previously reported instruments.[S2–S16]

XRD patterns were recorded on a diffractometer (MiniFlex 600, Rigaku; Cu Kα, λ = 1.5405 Å, 40 kV–15 mA) equipped with a high-speed 1-dimensional detector (D/teX Ultra, Rigaku). Diffraction data were collected in the range of 2θ = 10–90° in 0.02° steps with a scan rate of 20° min^{-1}. Thermogravimetry–differential thermal analysis (TG–DTA) profiles were recorded with a DTG-60 (Shimadzu, Japan). Samples (15 mg) were measured by TG-DTA under flowing air (200 mL min^{-1}). Attenuated total reflectance (ATR)-IR spectra were acquired at 298 K using a Shimadzu IRSpirit-T spectrometer equipped with a single bounce ATR cell (QATR-S, Shimadzu) and a triglycine sulfate detector. Diamond was used as an internal reflection (bevel of 45°). A total of 128 scans were recorded for each spectrum with a resolution of 2 cm^{-1}. Elemental analyses were performed by ICP-AES using a Shimadzu ICPS-8100 spectrometer. Nitrogen adsorption-

desorption isotherms were measured at −196 °C with a surface area and porosity analyzer (micromeritics TriStar II, Shimadzu). Prior to measurement, the samples were heated at 150 °C for 3 h under vacuum to remove physiosorbed water. The Brunauer-Emmett-Teller (BET) surface areas were estimated over the relative pressure (P/P_0) range of 0.05–0.30. TEM observations were conducted using a JEOL JEM2100F microscope operated at an accelerating voltage of 200 kV. After directly mixing Cu grids with samples, the Cu grids were collected and mounted on a stage. HAADF-STEM observations were conducted using an aberration corrected scanning transmission electron microscope (JEOL, JEM-ARM200F). The average oxidation states of Fe species were determined by iodometric titration using an auto titrator (Mettler Toledo, Easy Pro Titrator System). Samples (ca. 50 mg) was added to a mixed solution (20 mL) of 1 M HCl and 0.5 M KI, and the resulting solution was titrated with an aqueous solution of 0.05 M $Na_2S_2O_3$.[S17] XPS data were collected on a Shimadzu ESCA-3400HSE spectrometer with a standard Al Kα source (1486.6 eV) working at 10 kV and 25 mA. Samples were fixed on a double-stick carbon tape. The binding energies were calibrated using the C 1s band at 284.6 eV. The spectrum was fitted and evaluated by the XPS Peak 4.1 program, whereas the background was subtracted using the Shirley function. The deconvoluted Fe 2p spectrum of $La_{1-x}Sr_xFeO_{3-\delta}$ shows four peaks with binding energies around 709 and 711 eV, which correspond to Fe(III) and Fe(IV), respectively.[S18] The deconvoluted O 1s spectrum of $La_{1-x}Sr_xFeO_{3-\delta}$ shows three peaks with binding energies around 529 and 531 eV, which correspond to lattice oxygen and adsorbed oxygen, and adsorbed molecular water, respectively.[S4] H_2-TPR profiles were measured on a BEL Japan BELCAT-A equipped with thermal conductivity (TCD) and mass (Belmass) detectors. A 50 mg amount of sample was placed in a quartz cell and then heated at rate of 20 °C min^{-1} from room temperature to 500 °C under 5% H_2/Ar flow (50 mL min^{-1}). X-Ray absorption spectroscopy (XAS) analysis of the catalysts was performed at the BL01B1 beamline at SPring-8 (Japan Synchrotron Radiation Research Institute, Hyogo, Japan). The ring energy was 8 GeV, and the stored current was 99.5 mA. Fe K-edge (7.11 keV) X-ray absorption spectra were recorded using an Si(111) double-crystal monochromator. All spectra were recorded using the transmission method in quick-scan mode with ion chambers as detectors. Data reduction was performed using xTunes (Science & Technology Instruments).[S19]

Liquid-phase catalytic oxidation was performed with an organic synthesizer (ALHB-80& DTC-200HZ-3000, Techno Applications). Gas chromatography (GC) analyses for products in the liquid phase were performed on a Shimadzu GC-2025 equipped with a Stabilwax capillary column (internal diameter = 0.25 mm, length = 30

m, thickness = 0.25 μm) and a flame ionization detector. The retention times for acetone, *t*-BuOH, TBHP, and chlorobenzene (internal standard) are 4.0, 15.2, and 13.5 min, respectively, and no other significant product peaks were observed. For the analysis of products in the gas phase, a GL Science GC-3210 equipped with Gaskuropack 54 and MS-5A columns and a thermal conductivity detector was used. The retention times for isobutane, O_2, CO, CO_2, and N_2 (internal standard) are 15.0 (Gaskuropack 54), 3.8 (MS-5A), 6.8 (MS-5A), 2.2 (Gaskuropack 54), and 4.2 min (MS-5A), respectively. Mass spectroscopy (MS) measurements were recorded on a spectrometer (GCMSQP2010 SE, Shimadzu) equipped with an InertCap 17MS capillary column (internal diameter = 0.25 mm, length = 30 m, thickness = 0.25 μm) at an ionization voltage of 70 eV.

Synthesis of perovskite oxides

Perovskite oxide catalysts were synthesized by the sol–gel method using aspartic acid or malic acid in combination with metal acetates. A typical procedure for the iron-based $La_{0.8}Sr_{0.2}FeO_3$ perovskite catalyst was synthesized by the amino-acid aided method as described in refs S3, S4, S7–S9, S13–S15 and the details are as follows: $La(OAc)_3 \cdot 1.5H_2O$ (3.57 g, 10.4 mmol), $Sr(OAc)_2 \cdot 0.5H_2O$ (0.56 g, 2.6 mmol), $Fe(OAc)_2$ (13 mmol) and L-aspartic acid (39 mmol) were dissolved in water (500 mL). The brown solution was evaporated to dryness at 60 °C. The resulting brown powder was dried at 240 °C for 2 h under vacuum to give a pale brown powder. The precursor was calcined at 650 °C for 5 h in air to obtain $La_{0.8}Sr_{0.2}FeO_{3-\delta}$ (2.61 g, 87% yield). Other perovskite oxides such as $BaFeO_{3-\delta}$ (calcined at 750 °C),[S8,13] $SrMnO_3$ (calcined at 550 °C),[S15] $BaMnO_3$ (calcined at 550 °C),[S15] Mg_6MnO_8 (calcined at 550 °C)[S7] $LaNiO_3$ (calcined at 550 °C), $LaCoO_3$ (calcined at 550 °C), $La_{0.8}Ca_{0.2}FeO_{3-\delta}$ (calcined at 650 °C), $La_{0.8}Ba_{0.2}FeO_{3-\delta}$ (calcined at 650 °C) were also synthesized according to the literature procedures.

Procedure for catalytic oxidation of butanes

Catalytic oxidation was conducted in a 13 mL autoclave reactor with a Teflon vessel containing a magnetic stirring bar. A typical procedure for catalytic oxidation of isobutane was as follows: $La_{0.8}Sr_{0.2}FeO_{3-\delta}$ (0.1 g), isobutane (0.2 MPa, 3.2 mmol), $PhCF_3$ (2 mL), O_2 (0.25 MPa). The amounts of isobutane introduced in the reactor were confirmed by the direct weight measurement using an advanced-level analytical/precision balance (A&D, GX-1603A). The reaction solution was heated at 110 °C for 24 h. After checking the residual pressure in the autoclave reactor, the products in the gas phase was transferred into a sampling bag and analyzed by GC-TCD with gaskuropack 54 and molecular sieve 5A columns. The products in the liquid phase was analyzed by GC-FID with a Stabilwax column. Yields and selectivities were calculated on the C4-basis.

After the reaction, the catalyst was recovered by filtration, washed with $PhCF_3$ (20 mL) and methanol (20 mL), and then dried at 110 °C for 12 h before recycling. The amounts of surface Fe species were estimated assuming that the (001) plane is a surface structure because of the abundant population of Fe species (6.6 atoms per nm^2) on the (001) plane. The amounts of surface Fe were estimated using this hypothesis and the S_{BET} of $La_{0.8}Sr_{0.2}FeO_{3-\delta}$ to be 190 μmol·g^{-1}.

Procedure for catalytic decomposition of TBHP

Catalytic decomposition of TBHP was conducted in a 30 mL glass vessel containing a magnetic stirring bar. A typical procedure for catalytic decomposition was as follows: $La_{0.8}Sr_{0.2}FeO_{3-\delta}$ (50 mg), TBHP (0.5 mmol), $PhCF_3$ (2 mL), Ar (0.1 MPa), and an internal standard (naphthalene) were charged into the reaction vessel. The reaction solution was heated at 50 °C and periodically analyzed using GC.

References

S1. Williams, D. B.; Lawton, M., Drying of Organic Solvents: Quantitative Evaluation of the Efficiency of Several Desiccants. *J. Org. Chem.* **2010**, *75*, 8351–8354.

S2. Matsuda, A.; Obara, K.; Ishikawa, A.; Tsai, M.-H.; Wang, C.-H.; Lin, Y.-C.; Hara, M.; Kamata, K., Bismuth Phosphate Nanoparticle Catalyst for Direct Oxidation of Methane into Formaldehyde. *Catal. Sci. Technol.* **2023**, *13*, 5180–5189.

S3. Aihara, T.; Aoki, W.; Kiyohara, S.; Kumagai, Y.; Kamata, K.; Hara, M., Nanosized Ti-Based Perovskite Oxides as Acid-Base Bifunctional Catalysts for Cyanosilylation of Carbonyl Compounds. *ACS Appl. Mater. Interfaces* **2023**, *15*, 17957–17968.

S4. Shibata, S.; Kamata, K.; Hara, M., Stability Enhancement of Iron-based Perovskite Catalysts by A-site Substitution for Oxidative Transposition of α-Bromostyrene to Phenacyl Bromide. *ChemCatChem* **2022**, *14*, e202200395.

S5. Koutani, M.; Hayashi, E.; Kamata, K.; Hara, M., Synthesis and Aerobic Oxidation Catalysis of Mesoporous Todorokite-Type Manganese Oxide Nanoparticles by Crystallization of Precursors. *J. Am. Chem. Soc.* **2022**, *144*, 14090–14100.

S6. Kamata, K.; Kinoshita, N.; Koutani, M.; Aono, R.; Hayashi, E.; Hara, M., β-MnO_2 Nanoparticles as Heterogenous Catalysts for Aerobic Oxidative Transformation of Alcohols to Carbonyl Compounds, Nitriles, and Amides. *Catal. Sci. Technol.* **2022**, *12*, 6219–6230.

S7. Hayashi, E.; Tamura, T.; Aihara, T.; Kamata, K.; Hara, M., Base-Assisted Aerobic C-H Oxidation of Alkylarenes with a Murdochite-Type Oxide Mg_6MnO_8 Nanoparticle Catalyst. *ACS Appl. Mater. Interfaces* **2022**, *14*, 6528–6537.

S8. Shibata, S.; Kamata, K.; Hara, M., Aerobic Oxidative C=C Bond Cleavage of Aromatic Alkenes by A High Valency Iron-containing Perovskite Catalyst. *Catal. Sci. Technol.* **2021**, *11*, 2369–2373.

S9. Matsuda, A.; Tateno, H.; Kamata, K.; Hara, M., Iron Phosphate Nanoparticle Catalyst for Direct Oxidation of Methane into Formaldehyde: Effect of Surface Redox and Acid–base Properties. *Catal. Sci. Technol.* **2021**, *11*, 6987–6998.

S10. Yamaguchi, Y.; Aono, R.; Hayashi, E.; Kamata, K.; Hara, M., Template-Free Synthesis of Mesoporous β-MnO_2 Nanoparticles: Structure, Formation Mechanism, and Catalytic Properties. *ACS Appl. Mater. Interfaces* **2020**, *12*, 36004–36013.

S11. Hayashi, E.; Yamaguchi, Y.; Kita, Y.; Kamata, K.; Hara, M., One-pot Aerobic Oxidative Sulfonamidation of Aromatic Thiols with Ammonia by A Dual-functional β-MnO_2 Nanocatalyst. *Chem. Commun.* **2020**, *56*, 2095–2098.

S12. Hayashi, E.; Yamaguchi, Y.; Kamata, K.; Tsunoda, N.; Kumagai, Y.; Oba, F.; Hara, M., Effect of MnO_2 Crystal Structure on Aerobic Oxidation of 5-Hydroxymethylfurfural to 2,5-Furandicarboxylic Acid. *J. Am. Chem. Soc.* **2019**, *141*, 890–900.

S13. Shibata, S.; Sugahara, K.; Kamata, K.; Hara, M., Liquid-phase Oxidation of Alkanes with Molecular Oxygen Catalyzed by High Valent Iron-based Perovskite. *Chem. Commun.* **2018**, *54*, 6772–6775.

S14. Kamata, K.; Sugahara, K.; Kato, Y.; Muratsugu, S.; Kumagai, Y.; Oba, F.; Hara, M., Heterogeneously Catalyzed Aerobic Oxidation of Sulfides with a $BaRuO_3$ Nanoperovskite. *ACS Appl. Mater. Interfaces* **2018**, *10*, 23792–23801.

S15. Sugahara, K.; Kamata, K.; Muratsugu, S.; Hara, M., Amino Acid-Aided Synthesis of a Hexagonal $SrMnO_3$ Nanoperovskite Catalyst for Aerobic Oxidation. *ACS Omega* **2017**, *2*, 1608–1616.

S16. Kawasaki, S.; Kamata, K.; Hara, M., Dioxygen Activation by a Hexagonal $SrMnO_3$ Perovskite Catalyst for Aerobic Liquid-Phase Oxidation. *ChemCatChem* **2016**, *8*, 3247–3253.

S17. Rormark, L.; Wiik, K.; Stolen, S.; Grande, T., Oxygen Stoichiometry and Structural Properties of $La_{1-x}A_xMnO_{3\pm\delta}$ (A = Ca or Sr and $0 \le x \le 1$). *J. Mater. Chem.* **2002**, *12*, 1058–1067.

S18. Zhu, K.; Liu, H.; Li, X.; Li, Q.; Wang, J.; Zhu, X.; Yang, W., Oxygen Evolution Reaction over Fe site of $BaZr_xFe_{1-x}O_{3-\delta}$ Perovskite Oxides. *Electrochim. Acta* **2017**, *241*, 433–439.

S19. Asakura, H.; Yamazoe, S.; Misumi, T.; Fujita, A.; Tsukuda, T.; Tanaka, T., xTunes: A new XAS Processing Tool for Detailed and On-the-fly Analysis. *Radiat. Phys. Chem.* **2020**, *175*, 108270.
S20. Machkova, M.; Brashkova, N.; Ivanov, P.; Carda, J. B.; Kozhukharov, V., Surface Behavior of Sr-Doped Lanthanide Perovskites. *Appl. Surf. Sci.* **1997**, *119*, 127–136.
S21. Lyons, J. E.; Ellis, P. E.; Myers, H. K., Halogenated Metalloporphyrin Complexes as Catalysts for Selective Reactions of Acyclic Alkanes with Molecular Oxygen. *J. Catal.* **1995**, *155*, 59–73.
S22. Nizova, G. V.; Suss-Fink, G.; Shul'pin, G. B., Oxidations by the Reagent O_2-H_2O_2-vanadium Complex-pyrazine-2-carboxylic Acid. 8. Efficient Oxygenation of Methane and Other Lower Alkanes in Acetonitrile. *Tetrahedron* **1997**, *53*, 3603–3614.
S23. Sakaguchi, S.; Kato, S.; Iwahama, T.; Ishii, Y., An Efficient Aerobic Oxidation of Isobutane to *t*-Butyl Alcohol by *N*-Hydroxyphthalimide Combined with Co(II) Species. *Bull. Chem. Soc. Jpn.* **1998**, *71*, 1237–1240.
S24. Moore, K. T.; Horvath, I. T.; Therien, M. J., Mechanistic Studies of (Porphinato)iron-catalyzed Isobutane Oxidation. Comparative Studies of Three Classes of Electron-deficient Porphyrin Catalysts. *Inorg. Chem.* **2000**, *39*, 3125–3139.
S25. Rodionova, L. I.; Borisova, N. E.; Smirnov, A. V.; Ordomsky, V. V.; Moiseeva, A. A.; Pankratov, D. A., Binuclear Iron Complexes with Acyclic Schiff Bases Based on 4-*Tert*-butyl-2,6-diformylphenol: Synthesis, Properties, and Use in Catalytic Partial Oxidation of Isobutane. *Russ. Chem. Bull.* **2013**, *62*, 1201–1209.
S26. Yiu, S.-M.; Wu, Z.-B.; Mak, C.-K.; Lau, T.-C., $FeCl_3$-Activated Oxidation of Alkanes by [Os(N)O_3]. *J. Am. Chem. Soc.* **2004**, 126, 14921–14929.
S27. Mizuno, N.; Kamata, K.; Yamaguchi, K., Oxidative Functional Group Transformations with Hydrogen Peroxide Catalyzed by A Divanadium-substituted Phosphotungstate. *Catal. Today* **2012**, *185*, 157–161.
S28. Liu, X. H.; Yu, H. Y.; Huang, J. Y.; Su, J. H.; Xue, C.; Zhou, X. T.; He, Y. R.; He, Q.; Xu, D. J.; Xiong, C.; Ji, H. B., Biomimetic Catalytic Aerobic Oxidation of C–sp(3)–H Bonds under Mild Conditions using Galactose Oxidase Model Compound $Cu^{II}L$. *Chem. Sci.* **2022**, *13*, 9560–9568.

Table S1. Elemental analysis for $La_{1-x}Sr_xFeO_{3-\delta}$ based on ICP-AES [a]

x in $La_{1-x}Sr_xFeO_{3-\delta}$	elemental concentration (wt%) La	Sr	Fe	molar ratio (La:Sr:Fe)
0	60.3 (57.2)	0.0 (0)	23.3 (23.0)	1.04 : 0 : 1
0.2	46.1 (47.8)	7.0 (7.5)	26.2 (24.0)	0.71 : 0.17 : 1
0.4	36.9 (37.5)	16.0 (15.8)	26.8 (25.1)	0.55 : 0.38 : 1
0.6	27.0 (26.2)	26.1 (24.8)	29.4 (26.3)	0.37 : 0.57 : 1
0.8	13.4 (13.8)	33.7 (34.7)	29.2 (27.7)	0.18 : 0.74 : 1
1	0.0 (0)	34.1 (45.8)	22.9 (29.2)	0 : 0.95 : 1

[a]The values in the parenthesis are theoretical compositions when δ values are assumed to be zero.

Table S2. Effect of catalysts on the oxidation of isobutane with O_2[a]

entry	catalyst	yield		
		t-BuOH	TBHP	acetone
1	$BaFeO_{3-\delta}$	15.0	6.7	3.5
2	$SrFeO_{3-\delta}$	20.1	4.0	4.5
3	$LaFeO_3$	0.0	0.0	0.3
4	$La_{0.8}Sr_{0.2}FeO_{3-\delta}$	24.1	2.3	5.0
5	$La_{0.6}Sr_{0.4}FeO_{3-\delta}$	20.3	0.3	4.7
6	$La_{0.4}Sr_{0.6}FeO_{3-\delta}$	20.7	0.0	6.5
7	$La_{0.2}Sr_{0.8}FeO_{3-\delta}$	21.6	0.0	5.9
8	$La_{0.8}Ca_{0.2}FeO_{3-\delta}$	6.8	1.1	2.3
9	$La_{0.8}Ba_{0.2}FeO_{3-\delta}$	22.0	3.2	4.9
10	Fe_2O_3	0.1	0.0	0.0
11	Fe_3O_4	0.0	0.0	0.0
12	$Fe(OAc)_2$	0.3	0.0	0.1
13	$La(OAc)_3$	0.0	0.0	0.3
14	$Sr(OAc)_2$	0.0	0.0	0.0
15	$BaMnO_3$	0.7	0.0	0.0
16	$SrMnO_3$	0.3	0.0	0.0
17	Mg_6MnO_8	0.1	0.0	0.0
18	$LaNiO_3$	0.6	0.0	0.3
19	$LaCoO_3$	0.0	0.0	0.0
20	blank	0.1	0.0	0.5

[a] Reaction conditions: Catalyst (0.1 g), isobutane (0.2 MPa), O_2 (0.25 MPa), $PhCF_3$ (2 mL), 110 °C, 24 h.

Table S3. Recycling of $La_{0.8}Sr_{0.2}FeO_{3-\delta}$, $SrFeO_{3-\delta}$ and $BaFeO_{3-\delta}$ for the oxidation of isobutane with O_2[a]

entry	catalyst	yield (%)		
		t-BuOH	TBHP	acetone
1	$BaFeO_{3-\delta}$ (fresh)	15.0	6.7	3.5
2	$BaFeO_{3-\delta}$ (reuse)	–	–	–
3	$SrFeO_{3-\delta}$ (fresh)	20.1	4.0	4.5
4	$SrFeO_{3-\delta}$ (reuse)	–	–	–
5	$La_{0.8}Sr_{0.2}FeO_{3-\delta}$ (fresh)	24.1	2.3	5.0
6	$La_{0.8}Sr_{0.2}FeO_{3-\delta}$ (reuse 1st)	21.5	1.3	4.6
7	$La_{0.8}Sr_{0.2}FeO_{3-\delta}$ (reuse 2nd)	27.8	3.2	4.7

[a] Reaction conditions: Catalyst (0.1 g), isobutane (0.2 MPa), O_2 (0.25 MPa), $PhCF_3$ (2 mL), 110 °C, 24 h.

Table S4. Binding energy and fraction of iron-based perovskite oxides [a]

catalyst	binding energy of Fe 2p (eV)		binding energy of O 1s (eV)		
	Fe (IV)	Fe (III)	lattice oxygen	adsorbed oxygen	carbonate
$La_{0.8}Sr_{0.2}FeO_{3-\delta}$ -fresh	711.0 (27%)	709.6 (73%)	528.8 (59%)	531.2 (41%)	-
$La_{0.8}Sr_{0.2}FeO_{3-\delta}$ -recovered	711.0 (32%)	709.5 (68%)	528.6 (53%)	531.0 (47%)	-
$SrFeO_{3-\delta}$ -fresh	711.0 (72%)	709.9 (28%)	529.4 (50%)	531.6 (50%)	-
$SrFeO_{3-\delta}$ -recovered	711.0 (58%)	709.3 (42%)	528.7 (9%)	531.4 (61%)	533.6 (30%)
$BaFeO_{3-\delta}$ -fresh	711.0 (46%)	709.3 (54%)	529.2 (41%)	531.4 (59%)	-
$BaFeO_{3-\delta}$ -recovered	711.0 (35%)	709.7 (65%)	529.2 (35%)	531.4 (65%)	-

[a]The values in the parentheses are peak percentage.

Table S5. Effect of Sr substitution for the oxidation of isobutane with O_2 over $La_{1-x}Sr_xFeO_{3-\delta}$ [a]

entry	catalyst	yield (%)		
		t-BuOH	TBHP	acetone
1	$LaFeO_3$	0.1	0.0	0.0
2	$La_{0.8}Sr_{0.2}FeO_{3-\delta}$	8.8	0.2	1.6
3	$La_{0.6}Sr_{0.4}FeO_{3-\delta}$	13.3	0.5	2.0
4	$La_{0.4}Sr_{0.6}FeO_{3-\delta}$	12.7	0.7	1.9
5	$La_{0.2}Sr_{0.8}FeO_{3-\delta}$	9.3	0.4	2.0
6	$SrFeO_{3-\delta}$	0.1	0.0	0.0

[a] Reaction conditions: Catalyst (0.1 g), isobutane (0.2 MPa), O_2 (0.25 MPa), $PhCF_3$ (2 mL), 80 °C, 24 h.

Table S6. Catalytic oxidation of isobutane

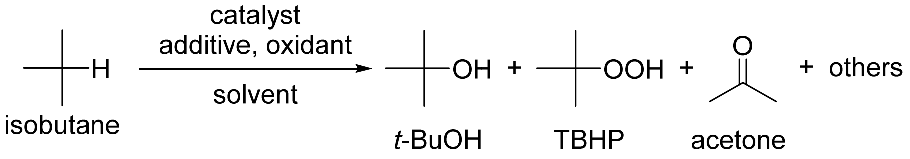

entry	catalyst	additive[a] (equiv.)	oxidant	solvent	temp. (°C)	time (h)	yield (%) (selectivity (%))				*t*-BuOH formation rate (mmol g-cat^{-1} h^{-1})	ref
							t-BuOH	TBHP	acetone	others		
1	$La_{0.8}Sr_{0.2}FeO_{3-\delta}$	–	O_2 (0.5 MPa)<br>O_2 (0.25 MPa)	$PhCF_3$<br>$PhCF_3$	110<br>60	24<br>24	43 (76)<br>10 (84)	4 (7)<br>1 (9)	8 (15)<br>0.7 (6)	CO, CO_2<br>CO, CO_2	0.57<br>0.13	This work
2[b]	Fe(TPPF$_{20}$)N_3	–	O_2 (0.7 MPa)	benzene	80	6	24 (89)	–	–	–	280	S21
3[b]	Fe(TPPF$_{20}$β-Br$_8$)Cl	–	O_2 (1.0)	neat (pressurized with N_2 (7.5MPa))	80	3	22 (78)	–	4 (15)	CO 0.3 (1)<br>CO_2 1.5 (5)	2700	S21
4[c]	TBA[VO_3]	PCA (4)	35 %H_2O_2<br>air (2.5 MPa)	CH_3CN	75	2.5	16[d] (44)	6[d] (15)	2[d] (4)	IBHP 3[d] (7)<br>IBOH 3[d] (7)<br>IBA 6[d] (15)	830	S22
5[e]	Co(OAc)$_2$	NHPI (40)	air (1 MPa)	benzonitrile	100	8	81 (86)	–	14 (15)	–	160 (based on Co)<br>6.2 (based on NHPI)	S23
6[f]	$(C_3F_7)_4$PFe-(py)$_2$	–	O_2 (0.83 MPa)	benzene	80	21	14 (81)	2 (12)	1 (8)	CO, CO_2	55	S24
7[g]	binuclear iron complex	TBHP (130)	air (3.5 MPa)	*t*-BuOH	130	6	7 (75)	2 (25)	–	–	160	S25
8[h]	TBA[Os(N)(O)$_3$]	$FeCl_3$ (8)	Cl_2pyO	CH_2Cl_2/AcOH	60	0.5	38[d] (79)	–	–	10[d] (21)	150	S26
9	TBA$_4$[γ-PW$_{10}$O$_{38}$V$_2$(μ-OH)(μ-O)]	$HClO_4$ (1)	30% H_2O_2	CH_3CN/*t*-BuOH	60	5	29[d] (n.d.)	–	–	–	0.62	S27
10[i]	Cu^{II}L	NHPI	O_2 (1 MPa)	CH_3CN	70	7	60 (80)	–	15 (20)	–	19 (based on Co)<br>13 (based on NHPI)	S28

[a] The amounts of additive with respect to that the catalyst used. [b] TPPF$_{20}$: meso-tetrakis(pentafluorophenyl)porphyrin dianion. TPPF$_{20}$β-Br$_8$: meso-tetrakis(pentafluorophenyl)-β-octabromoporphyrin dianion. [c] TBA: tetra-*n*-butyl ammonium. PCA: Pyrazine-2-carboxylic acid, IBHP: Isobutyl hydroperoxide. IBOH: Isobutyl alchol. IBA: Isobutyraldehyde. [d] The yield was determined based on the amounts of oxidants used (H_2O_2, pyridine N-oxide). [e] NHPI: . [f] [$(C_3F_7)_4PH_2$]: 5,10,15,20-tetrakis(heptafluoropropyl)porphyrin. [g] Binuclear iron complex : (μ$_2$-methoxy)-[*S*,*S*-μ$_2$-2,6-bis(1-carboxy-2-imidazol-4-yl-ethyliminomethyl)-4-tert-butylphenolato]diiron(III) dichloride. [h] ClpyO: 2,6-dicholoropyridine *N*-oxide. 2-Choloro-2-methylpropane. [i] L = : *N*,*N'*-bis(3,5-di-*tert*-butyl-2-hydroxyphenyl)-1,2-phenylenediamine.

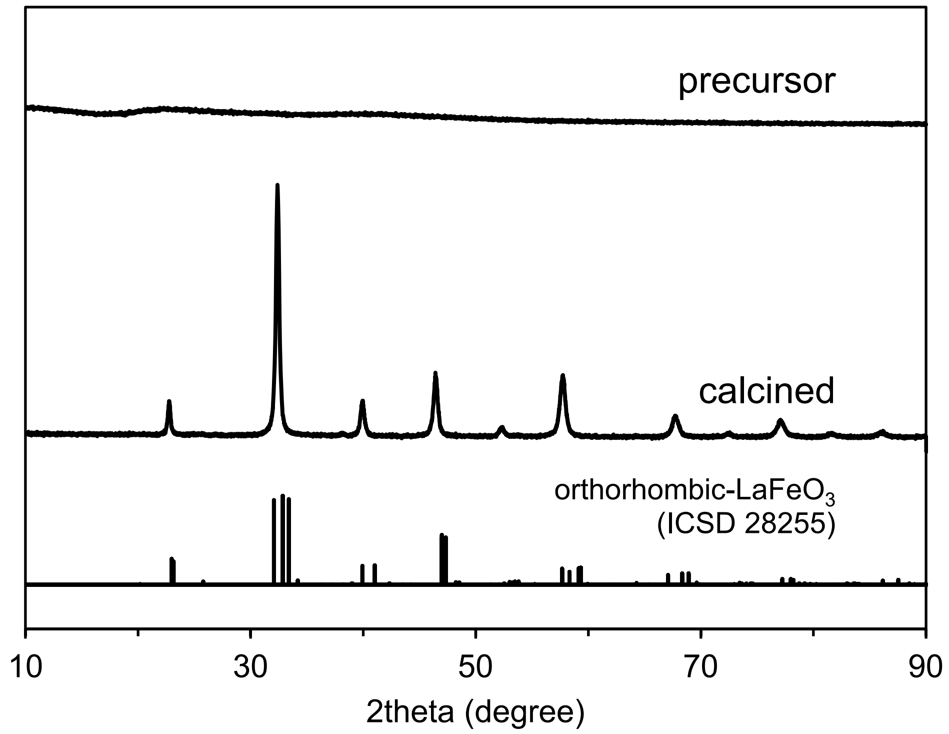

Figure S1. XRD patterns for $La_{0.8}Sr_{0.2}FeO_{3-\delta}$ precursor and calcined $La_{0.8}Sr_{0.2}FeO_{3-\delta}$.

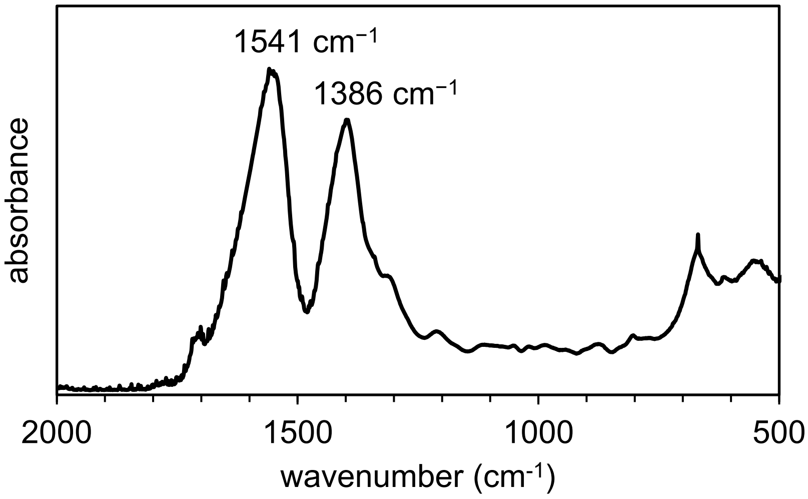

Figure S2. ATR-IR spectrum of $La_{0.8}Sr_{0.2}FeO_{3-\delta}$ precursor.

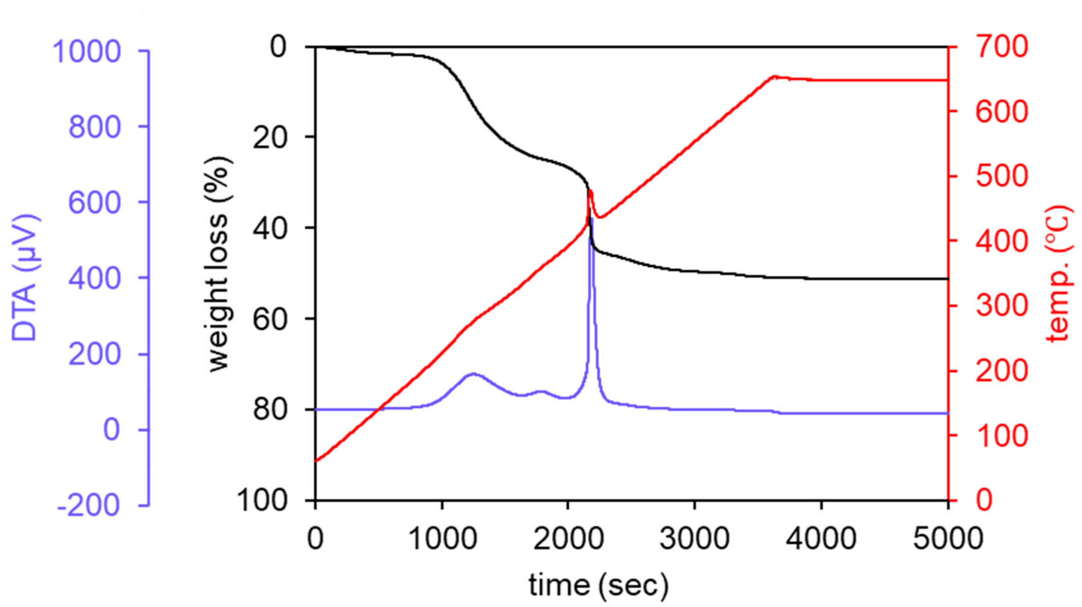

Figure S3. Thermogravimetry-differential thermal analytical (TG-DTA) curves for $La_{0.8}Sr_{0.2}FeO_{3-\delta}$ precursor.

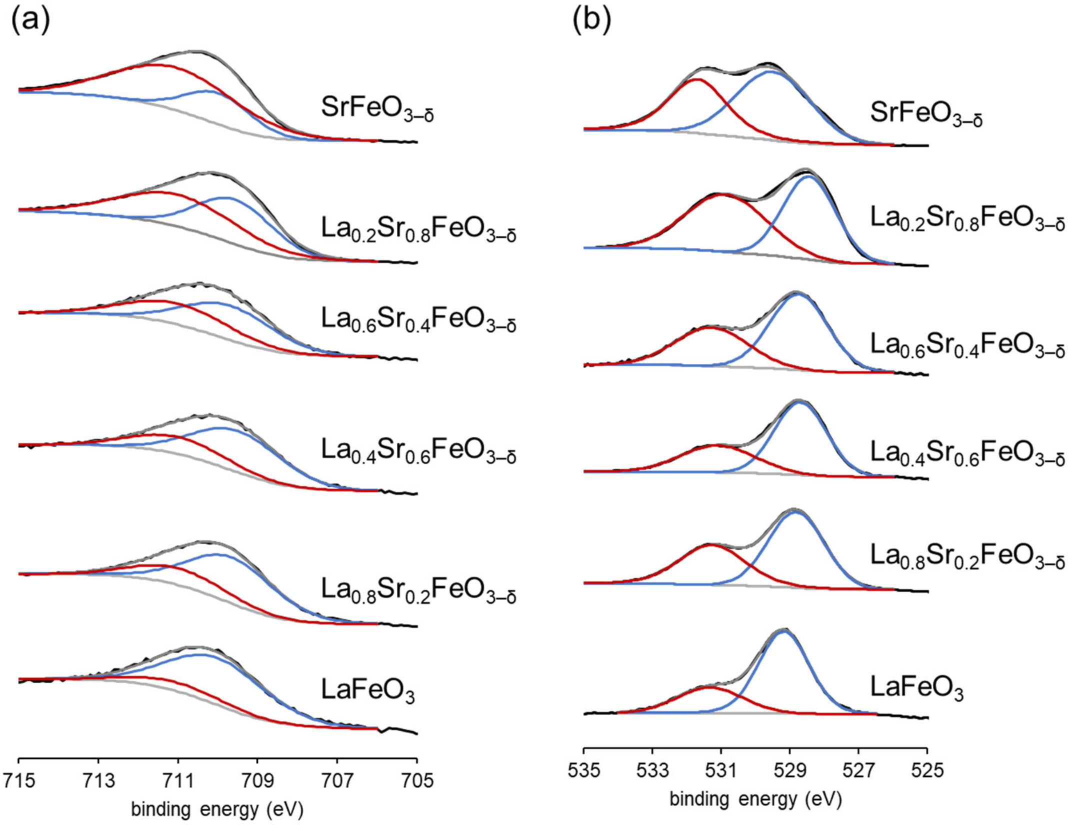

Figure S4. (a) Fe 2p and (b) O 1s XPS spectra of $La_{1-x}Sr_xFeO_{3-\delta}$.

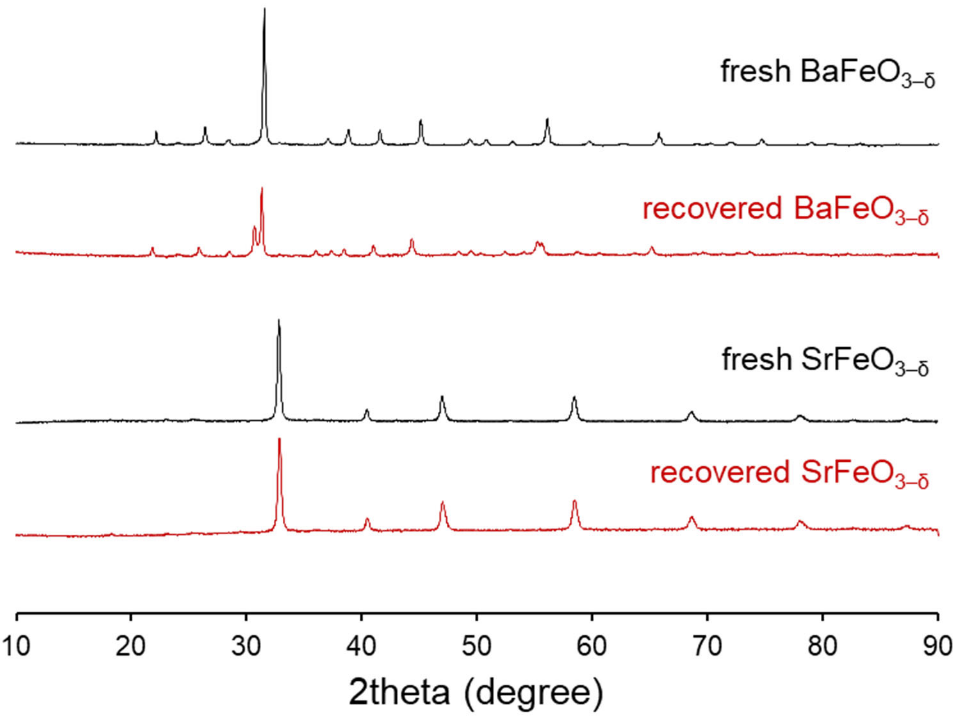

Figure S5. XRD patterns of fresh and recovered $SrFeO_{3-\delta}$ and $BaFeO_{3-\delta}$ for the oxidation of isobutane with O_2. Reaction conditions are the same as those in Figure 5.

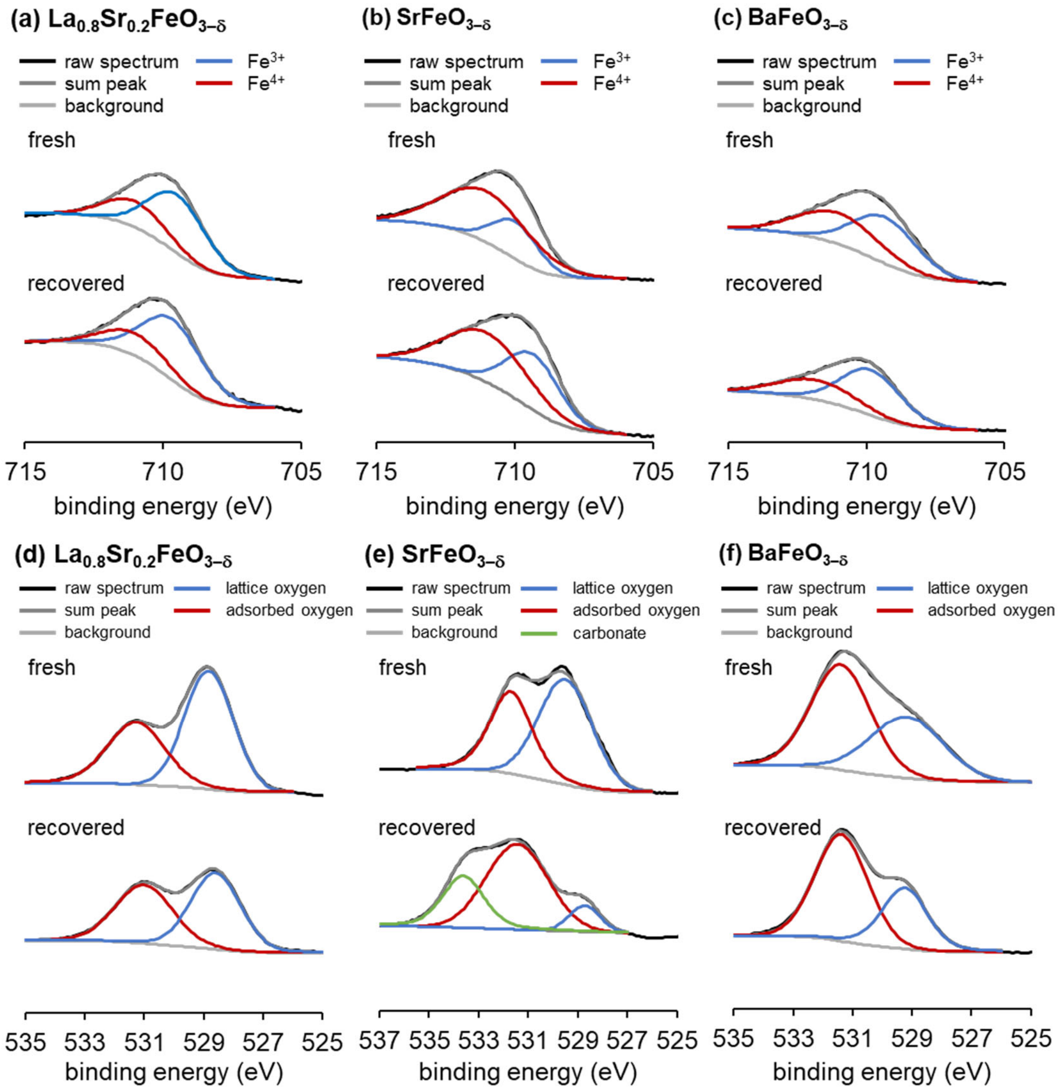

Figure S6. (a-c) O1s and (d-f) Fe 2p XPS spectra of fresh and recovered $La_{0.8}Sr_{0.2}FeO_{3-\delta}$, $SrFeO_{3-\delta}$ and $BaFeO_{3-\delta}$ for the oxidation of isobutane with O_2. Reaction conditions are the same as those in Figure 5.

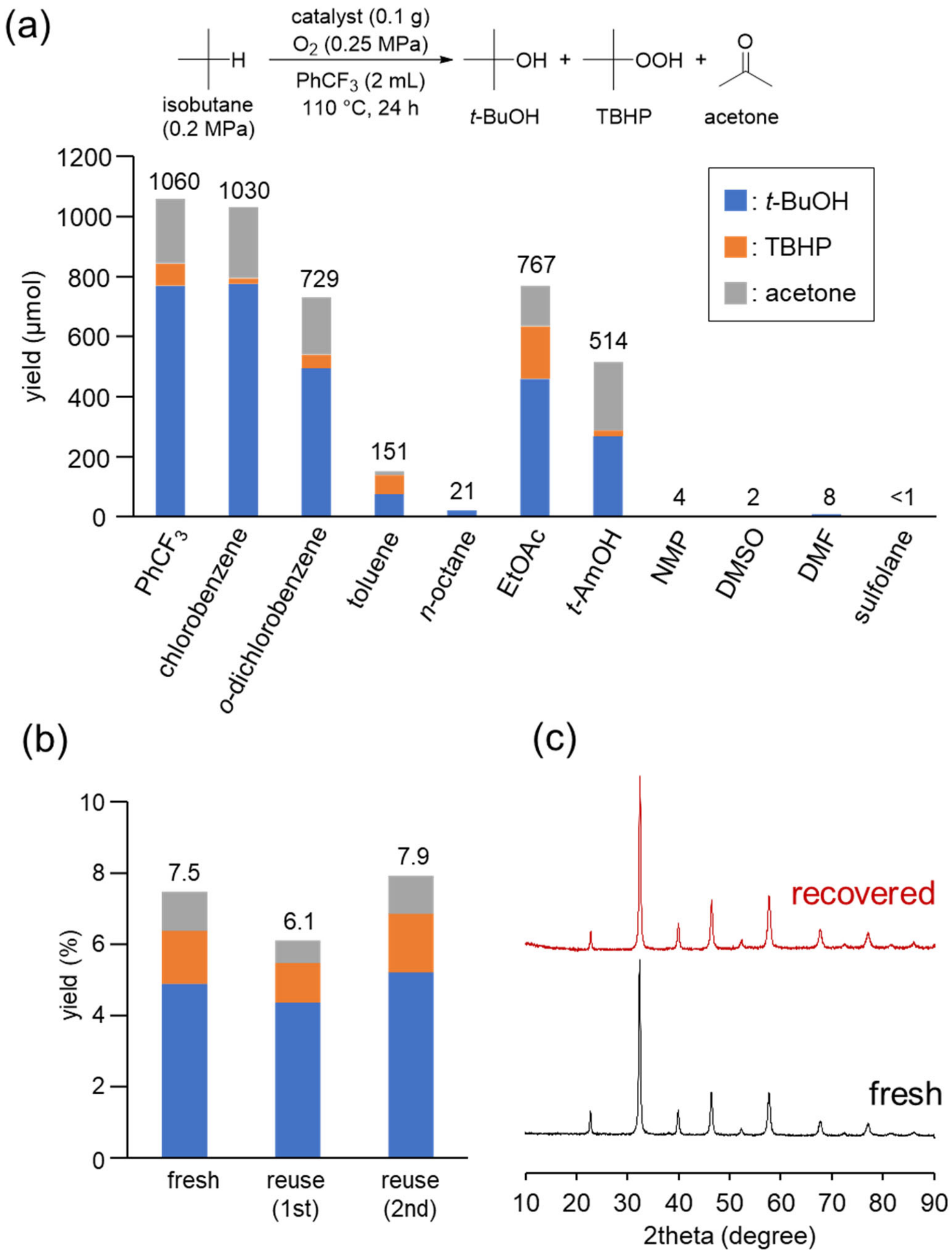

Figure S7. (a) Effect of solvents on the oxidation of isobutane with O_2 over $La_{0.8}Sr_{0.2}FeO_{3-\delta}$. Reaction conditions: $La_{0.8}Sr_{0.2}FeO_{3-\delta}$ (0.1 g), isobutane (0.2 MPa), O_2 (0.25 MPa), solvent (2 mL), 110 °C, 24 h. The amounts of soluble isobutane was strongly dependent on the types of solvents; thus, the y-axis was shown as the amounts of products. (b) Recycling of $La_{0.8}Sr_{0.2}FeO_{3-\delta}$ for the oxidation of isobutane in EtOAc with O_2. (c) XRD patterns of fresh and recovered $La_{0.8}Sr_{0.2}FeO_{3-\delta}$ after the oxidation in EtOAc.

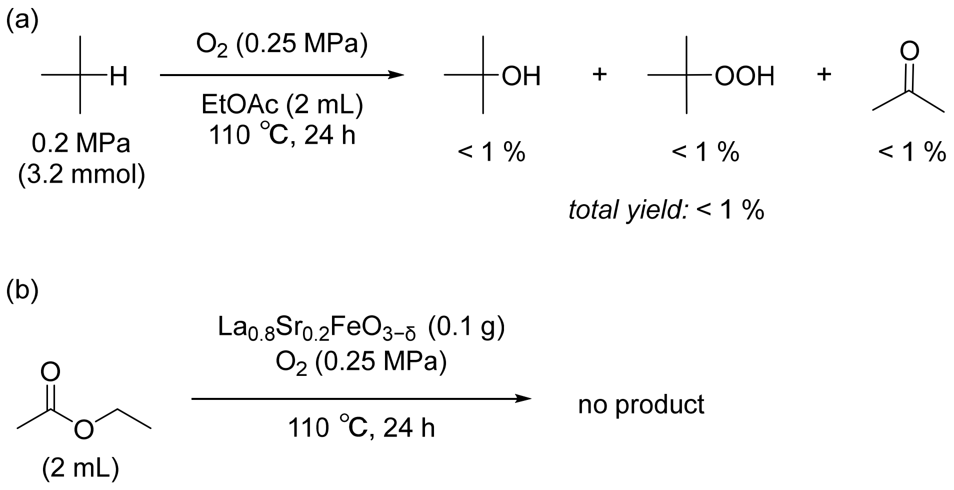

Figure S8. Blank experiments in the absence of (a) $La_{0.8}Sr_{0.2}FeO_{3-\delta}$ catalyst and (b) isobutane using EtOAc as solvent.

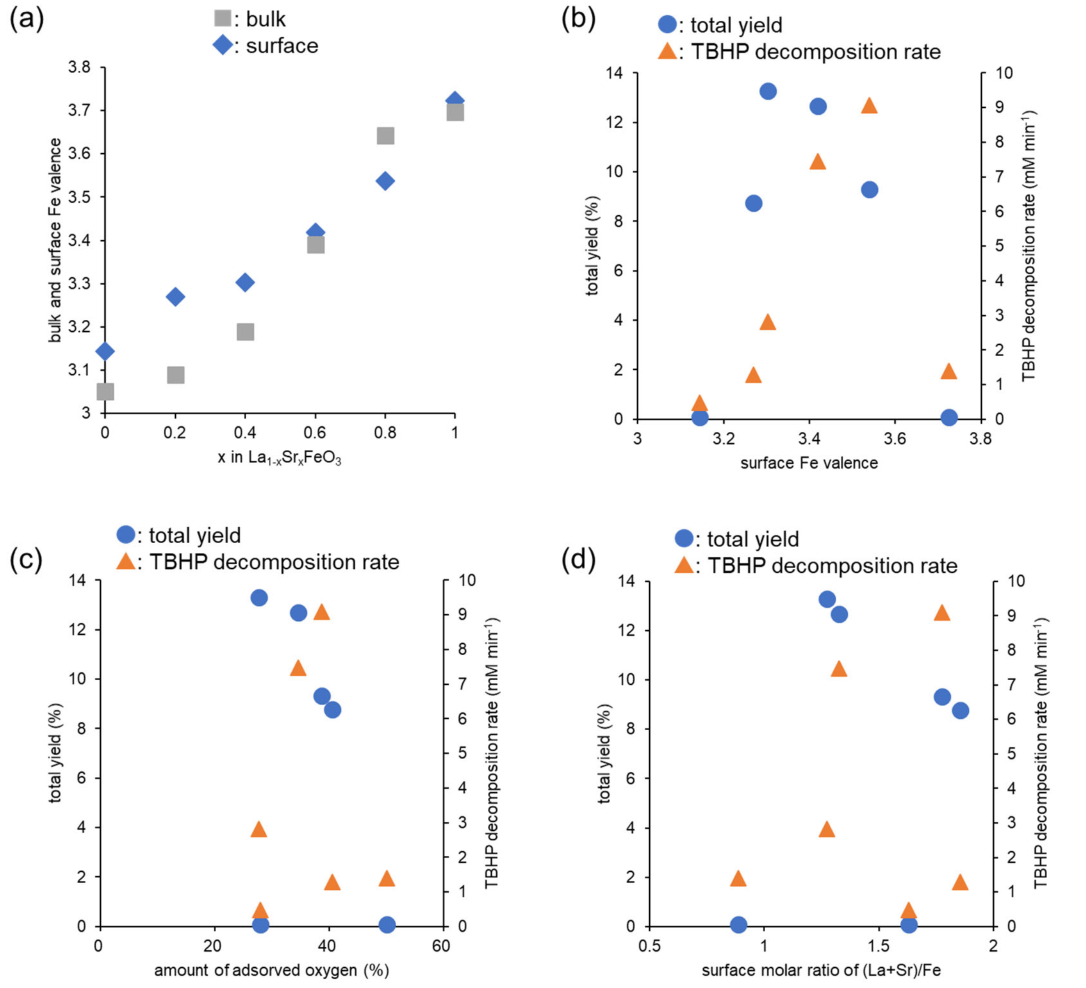

Figure S9. (a) Fe valence determined by iodometry and XPS. Plots of reactivity against (b) surface Fe valence, (c) amount of adsorbed oxygen, and (d) surface molar ratio of (La+Sr)/Fe.

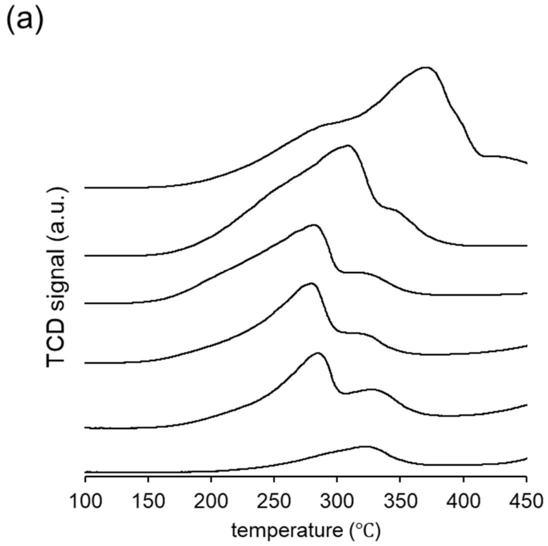

Figure S10. (a) H_2-TPR profiles of $La_{1-x}Sr_xFeO_{3-\delta}$ and (b) dependence of onset reduction temperature on the Sr content.
